# Tellurium-Terminated MXene Synthesis via One-Step Tellurium Etching

**DOI:** 10.1007/s40820-025-01875-1

**Published:** 2025-08-11

**Authors:** Guoliang Ma, Zongbin Luo, Hui Shao, Yanbin Shen, Zifeng Lin, Patrice Simon

**Affiliations:** 1 College of Materials Science and Engineering, Sichuan University, Chengdu, 610065 People’s Republic of China; 2 Lab, CAS Center for Excellence in Nanoscience, Suzhou Institute of Nano-Tech and Nano-Bionics (SINANO), Chinese Academy of Sciences (CAS), Suzhou, 215123 People’s Republic of China; 3 CIRIMAT, Université de Toulouse, CNRS, Toulouse, France

**Keywords:** Te-terminated MXene, Elemental tellurium etching, Sodium-ion storage, High-rate performance

## Abstract

With the rapid development of two-dimensional MXene materials, numerous preparation strategies have been proposed to enhance synthesis efficiency, mitigate environmental impact, and enable scalability for large-scale production. The compound etching approach, which relies on cationic oxidation of the A element of MAX phase precursors while anions typically adsorb onto MXene surfaces as functional groups, remains the main prevalent strategy. By contrast, synthesis methodologies utilizing elemental etching agents have been rarely reported. Here, we report a new elemental tellurium (Te)-based etching strategy for the preparation of MXene materials with tunable surface chemistry. By selectively removing the A-site element in MAX phases using Te, our approach avoids the use of toxic fluoride reagents and achieves tellurium-terminated surface groups that significantly enhance sodium storage performance. Experimental results show that Te-etched MXene delivers substantially higher capacities (exceeding 50% improvement over conventionally etched MXene) with superior rate capability, retaining high capacity at large current densities and demonstrating over 90% capacity retention after 1000 cycles. This innovative synthetic strategy provides new insight into controllable MXene preparation and performance optimization, while the as-obtained materials hold promises for high-performance sodium-ion batteries and other energy storage systems.

**Supplementary Information:**

The online version contains supplementary material available at 10.1007/s40820-025-01875-1.

## Highlights

- A novel and efficient Te etching method for the preparation of Te-functionalized MXene materials is presented
- This simple etching method enables the processing of V- and Nb-based MAX phases and demonstrates potential for large-scale production.
- V_2_CTe_*x*_ MXene has a sodium storage capacity of up to 247 mAh g^−1^ and maintains 216 mAh g^−1^ at 23 C.

**Supplementary Information:**

The online version contains supplementary material available at 10.1007/s40820-025-01875-1.

## Graphical abstract

**Figure:**
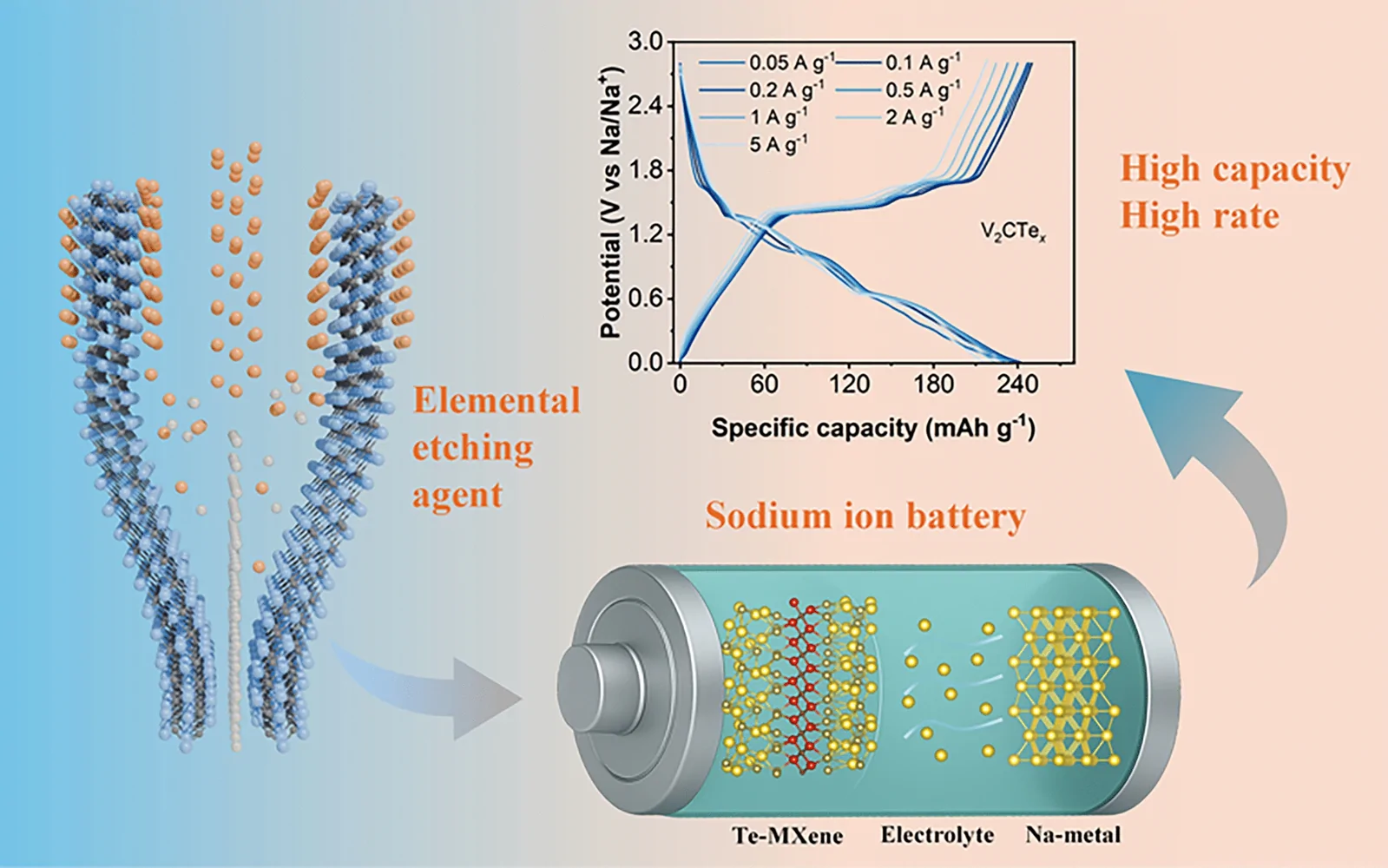

## Introduction

As nanomaterials research continues to advance rapidly, two-dimensional (2D) materials have become a prominent focus in materials science, owing to their unique layered structures and exceptional physicochemical properties [1, 2]. In particular, MXene-defined by the general formula M_*n*+1_X_*n*_T_*x*_—where M is a transition metal (e.g., Ti, V, Zr, Cr), X is carbon and/or nitrogen, and T_*x*_ represents surface terminations—is a class of transition-metal carbides, nitrides, and carbonitrides with a characteristic layered morphology [3, 4]. Their diverse chemical compositions, high electronic conductivity, and highly tailorable surface functional groups have garnered widespread attention [5, 6]. Since the initial discovery of MXene at 2011 [7], their morphologies have evolved from simple colloidal suspensions to powders and quantum dots [8], offering expanded chemical diversity [9] and extended applications in energy storage [10–17], catalysis [18], electromagnetic shielding [19], sensing [20], and biomedicine [21]. The unique structural and surface chemical features of MXene underpin these remarkable applications, emphasizing the importance of developing effective preparation methods that enable precise control over their surface chemistry [9, 22].

To date, various etching strategies have been developed for MXene synthesis, including wet chemical and high-temperature methods. The conventional approach employs concentrated hydrofluoric acid (HF) to selectively remove the A-layer from MAX phases, enabling the first successful synthesis of MXene [7, 23]. However, this method poses serious safety and environmental risks and typically yields MXene with mixed -F and -OH terminations that are difficult to precisely control. Compared with direct HF etching, this approach is relatively mild and safer, while the concurrent insertion of metal cations and water molecules between layers increases the interlayer spacing and reduces defects. Nevertheless, fluorine-containing reagents remain integral to this process, making it challenging to eliminate -F groups, with potential implications for both environmental impact and the intrinsic properties of MXene [24]. Later, a fluorine-free molten-salt Lewis acid etching method has gained interest [25, 26]. By exploiting molten chlorides (e.g., CuCl_2_) under an inert atmosphere, the A-site element in MAX phases can be selectively replaced, yielding MXene with primarily Cl terminations [27]. Although this process avoids toxic fluorides and broadens the range of surface terminations [28], it requires high temperatures in a strongly Lewis-acidic environment, often introducing significant structural defects. Recently, emerging vapor-phase etching approaches have been developed by introducing gaseous elemental halogens (Cl_2_, Br_2_, I_2_) at elevated temperatures in sealed conditions to yield MXene terminated with -Cl, -Br, or -I groups [29]. However, such gas-phase reactions require strict operating conditions and specialized equipment, limiting large-scale deployment. Furthermore, several intriguing environmentally friendly methods for preparing MXene have been reported, such as thermal reduction and ultraviolet-induced etching [30, 31]. Nevertheless, these methods have not comprehensively explored the universal applicability concerning MAX phase precursors. Hence, current etching methods inevitably involve trade-offs in efficiency, safety, and control over the resultant surface terminations, often falling short in achieving the flexible, on-demand synthesis of MXene. Existing strategies typically fix the species of terminal groups to those derived from the chosen reagents, highlighting the challenge of tailoring surface chemistry to optimize MXene performance [32]. To address these shortcomings, “elemental etching” was recently proposed as a potential new pathway, aiming to leverage direct reactions between elemental reagents and MAX precursors to remove the A-layer and introduce targeted functional groups in situ. Compared with conventional fluoride-based acidic etching, elemental etching promises greater flexibility for modulating functional groups, enhanced safety by avoiding strong corrosive acids, and clear thermodynamic driving forces (e.g., forming stable by-products) that could, in principle, enable fine-tuned control over MXene surface chemistry. Although research in this direction remains in its infancy—with limited systematic process optimization and performance evaluation, the approach offers exciting opportunities for overcoming the limitations of traditional techniques.

Herein, we propose a Te-based elemental etching strategy for synthesizing MXene from MAX phases. We find that direct reaction between Te and the MAX precursor at elevated temperatures simultaneously enables selective removal of the A-layer (Al in this work) and in situ formation of Te-containing surface groups on the resultant MXene. Compared with conventional multi-step processes, our single-step strategy eliminates the need for additional surface functionalization steps, thereby greatly simplifying the synthesis. In contrast to halogen-based etching methods, where halogen elements are typically in the gaseous phase during the reaction, Te exists in the liquid phase under the reaction conditions, offering lower processing requirements and improved controllability. Moreover, Te functionalization not only expands the interlayer spacing and reduces structural defects but also preserves high electrical conductivity owing to the relatively low electronegativity of Te. As a result, the as-prepared MXene exhibits excellent electrochemical performance and structural stability. Moreover, the Te-based etching process delivers a high yield of approximately 77% and exhibits excellent batch-to-batch reproducibility. The method is readily scalable and applicable to the synthesis of a wide range of MXene from different MAX phases. Although Te is a relatively less abundant element, it can be sourced from industrial by-products, and its consumption in this process remains well-controlled. Moreover, the utilized Te can be readily recovered via electrochemical reduction, enabling sustainable material recycling for potential scale-up. Benefiting from synergistic improvements in structure and surface properties, Te-terminated MXene deliver outstanding sodium-ion storage performance. Notably, V_2_CTe_*x*_ exhibits an excellent capacity of 247 mAh g^−1^, retaining 216 mAh g^−1^ at a 23 C rate. Overall, our Te-based elemental etching method represents a new avenue for the controllable synthesis of high-performance MXene, holding significant promise for both fundamental research and practical energy storage applications.

## Experimental Section

### Synthesis of Te-MXene

The preparation of Te-based MXene materials primarily involves mixing various MAX phases with elemental Te. Then, they are held in an Ar gas tube furnace at 700 °C for 1–2 h. Detailed preparation process can be found on supporting materials.

### Preparation of Te-MXene Anode

The electrode was prepared by mixing the MXene material, conductive additive (acetylene black), and binder (PVDF) in a weight ratio of 8:1:1. The mixture was homogenized using N-methyl-2-pyrrolidone (NMP) as the solvent and then coated onto carbon-coated copper foil. The coated foil was dried at 110 °C for 12 h and subsequently punched into circular disks (12 mm in diameter) for use as electrodes. The active material loading on the MXene electrode was approximately 1.0–1.5 mg cm^−2^. The electrolyte volume used per cell was approximately 110 μL.

### Theoretical Calculation

The theoretical calculations based on density functional theory (DFT) were performed using the DS-PAW software package integrated within Device Studio [33]. Detailed preparation process can be found on supporting materials.

### Electrochemical Test

All electrochemical measurements were taken using CR2032 coin cells. For the counter electrode, lithium metal foil or sodium metal foil was used. In lithium-ion battery tests, glass fiber (GF/A) was employed as the separator, while GF/D was used in sodium-ion battery tests. The electrolyte for lithium-ion batteries was 1 m LiPF_6_ in a mixture of ethylene carbonate (EC) and dimethyl carbonate (DMC), and for sodium-ion batteries, 1 m NaPF_6_ in 1,2-dimethoxyethane (DME) was used. All cells underwent activation treatment at a current density of 20 mA g^–1^ before rate performance, long-cycle, and full-cell tests. For half-cell tests, the voltage range was set between 0.01 and 2.8 V. The assembly of all cells was performed in an argon-filled glove box to ensure an inert atmosphere. Electrochemical tests were primarily conducted using a Neware eight-channel battery testing system (CT-4008 T). All tests were performed in a temperature-controlled chamber (MJS-DZ250, Nanjing Mojiesi) maintained at 27 °C.

## Results and Discussion

### Principles of Te-MXene Material Preparation

**Fig. 1:**
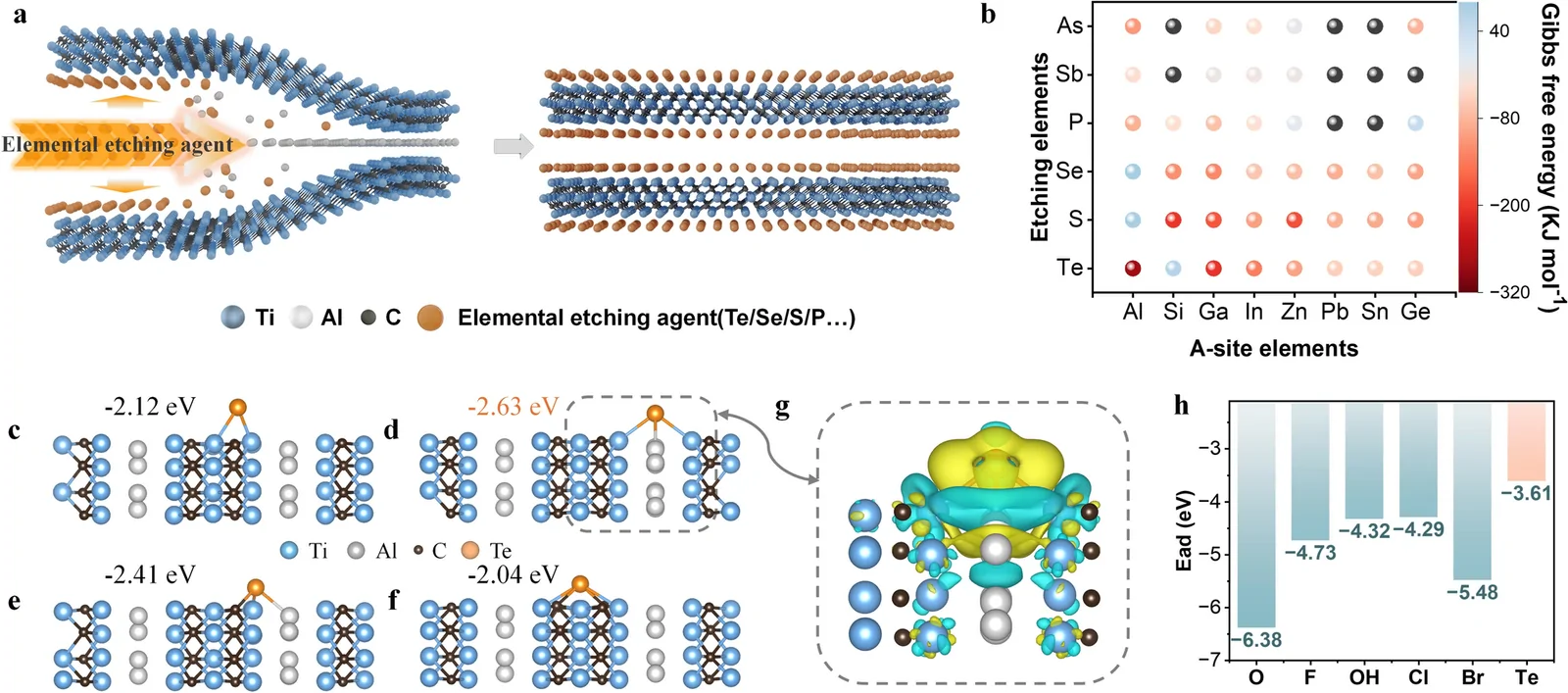
Principles of MXene synthesis.** a** Schematic illustration of MXene materials prepared by element etching. **b** Gibbs free energies of reaction for various A-site elements (Al, Si, Ga, In, Zn, Pb, Sn, Ge) reacting with Te, S, Se, P, Sb, and As at 700 °C. Adsorption energies of Te at distinct sites of Ti_3_AlC_2_, including **c** top-C, **d** top-Al, **e** top-Ti1, **f** top-Ti2. **g** Theoretical calculation of differential charge at the Al site following Te adsorption on Ti_3_AlC_2_. **h** Adsorption energies of different functional groups of Ti_3_C_2_

A schematic illustration of the proposed elemental etching reaction (X = Te/Se/S/P…) for selectively converting MAX into MXene is depicted in Fig. 1a. Distinct from conventional compound-based etching systems, this method features a direct redox reaction between the elemental etchant X and the A-site element in a sealed environment, resulting in the formation of X_*x*_A_*y*_ compounds and enabling the selective removal of the A-site species. Meanwhile, the etching element X interacts with the exposed transition-metal (M) atoms on the MXene surface, leading to the formation of X-rich surface terminations. This elementary one-step etching process provides a rational design principle for the precise modulation of MXene surface chemistry. To assess the thermodynamic feasibility, we first constructed a Gibbs free energy (ΔG) model for reactions between elemental X and typical A-site elements (Al, Ga, In, Zn, Pb, Sn, Ge) at 700 °C (Fig. 1b). For Te, ΔG is markedly negative for most A-site element candidates (e.g., ΔG =  − 276.96 kJ mol^−1^ for Al), indicating a highly spontaneous reaction. Temperature-dependent calculations (Figs. 1b and S1) reveal that for the Al-containing MAX system, ΔG is largest (most negative) at higher temperatures, consistent with the preferential removal of Al in molten Lewis salts. DFT calculations for the model Ti_3_AlC_2_ system (Fig. 1c, d, e, f and g) indicate that the Te atom strongly adsorbs near the Al site (with an adsorption energy of − 2.63 eV), accompanied by pronounced charge transfer. This strong electronic interaction favors fast redox coupling and likely leads to the formation of intermediate Te_*x*_Al_*y*_ phases, ultimately driving selective Al removal. Notably, while Te exhibits a lower adsorption energy on Ti_3_C_2_ (− 3.61 eV) compared with –OH (− 4.32 eV) or –O (− 6.38 eV), as shown in Fig. 1h. This indicates that to get Te-terminated MXene conventional etching routes would require additional post-treatment steps to displace original functional groups (e.g., high-temperature substitution [34]), since the formation of Te groups is kinetically unfavored in situ. Using elemental Te as the etching reagent could direct formation of the desired Te functional groups during the etching process, highlighting advantages in both simplicity and controllability.

### Te-MXene Material Characterization

**Fig. 2:**
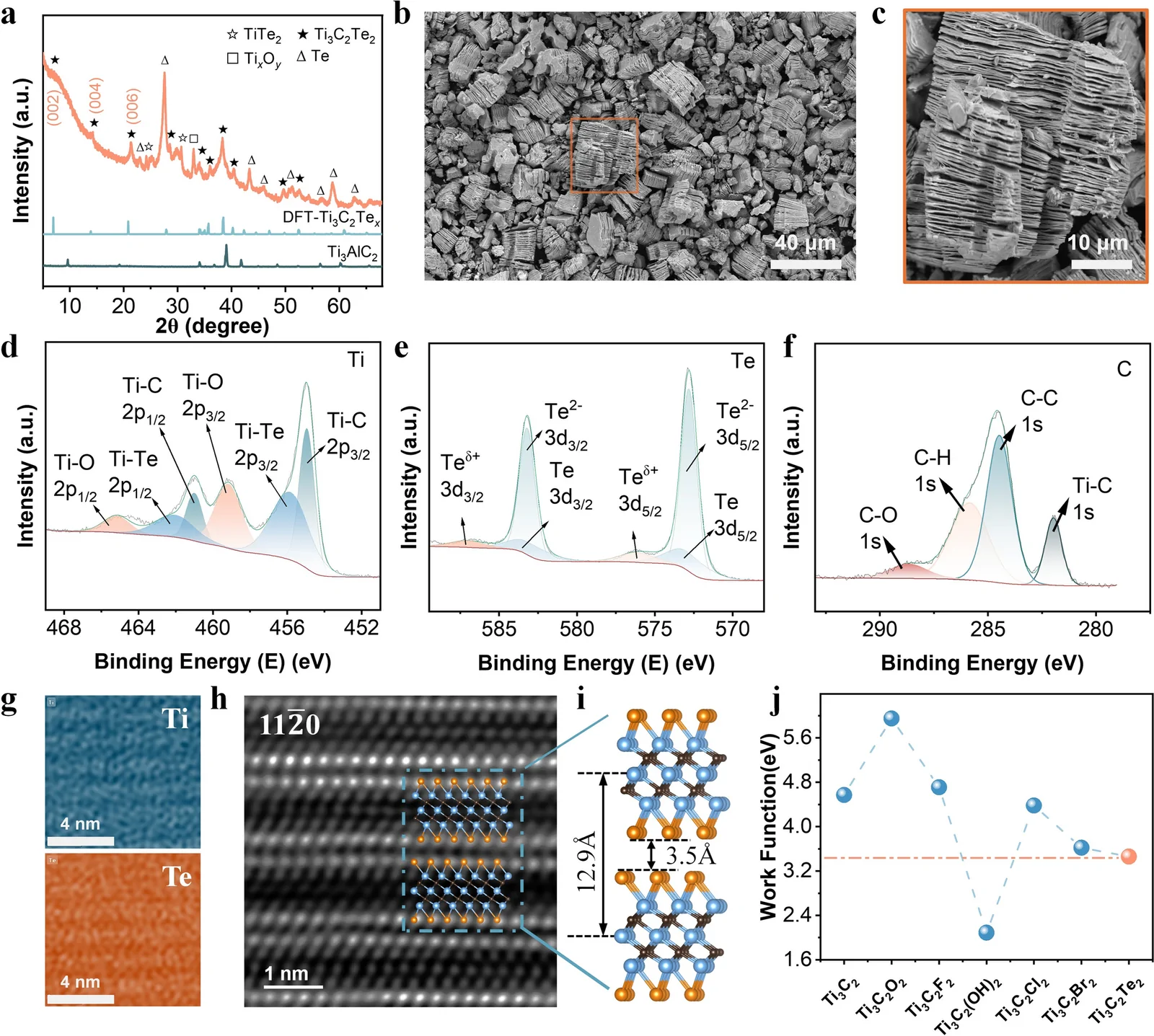
Ti_3_C_2_Te_*x*_ MXene characterization.** a** XRD patterns of Ti_3_AlC_2_ MAX phase and Ti_3_C_2_T_*x*_ obtained from experimental characterization and theoretical calculation. **b-c** SEM images of Ti_3_C_2_Te_*x*_. XPS spectra of Ti_3_C_2_Te_*x*_ MXene highlighting **d** Ti, **e** Te, and **f** C. **g** STEM-based elemental mapping revealing Ti and Te distributions. **h** Spherical aberration-corrected TEM image along [11 $\overline{2 }$ 0] zone axis, **i** Schematic representation of the atomic structure. **j** Calculated work functions of Ti_3_C_2_ with different functional groups

We first systematically investigated the reaction mechanism and process optimization for Te-mediated conversion of Ti_3_AlC_2_ into MXene. To systematically optimize the synthesis conditions, we investigated a range of parameters, including the Te/Ti_3_AlC_2_ molar ratio (1:1–4:1), reaction temperature (500–900 °C), and holding time (0–6 h). Solid-state reactions under Ar revealed incomplete removal of Al for Te/Ti_3_AlC_2_ ratios below 3:1, as evidenced by the characteristic (002) and (104) diffraction peaks at 9.6° and 39.1°, respectively (Fig. S2). Excess Te (4:1) resulted in secondary phase formation of TiTe_2_. Consequently, 3:1 provided optimal stoichiometric balance, ensuring complete etching while minimizing by-product formation. Temperature dependence (Fig. S3a) further indicated that effective etching occurs above 452 °C (the melting point of Te), suggesting a low reaction barrier. Balancing reaction kinetics and efficiency, 700 °C was identified as the optimal temperature for producing high-quality MXene. Moreover, as shown in Fig. S3b, excellent etching performance can be achieved even with a very short reaction time, indicating highly favorable and rapid reaction kinetics. Figure 2a presents the X-ray diffraction (XRD) pattern of the final product obtained at a Te/Ti_3_AlC_2_ molar ratio of 3:1 and 700 °C, after HCl washing, alongside that of the Ti_3_AlC_2_ precursor for comparison. The absence of characteristic Ti_3_AlC_2_ peaks indicates the successful removal of Al, while the pronounced (00* l*) reflections confirm the formation of MXene layers. The (002) peak of the Te-functionalized Ti_3_C_2_Te_*x*_ phase is observed at 6.99°, with a derived interlayer spacing of 12.64 Å. The attenuated intensity of the (002) reflection suggests weakened periodicity along the c-axis, commonly observed in MXene with larger or more complex functional groups. Compared to conventional Ti_3_C_2_Cl_2_ and Ti_3_C_2_Br_2_, the Te-terminated Ti_3_C_2_Te_*x*_ exhibits an expanded interlayer spacing, which facilitates enhanced ion diffusion and improved electrolyte accessibility (Fig. S4). Moreover, Ti_3_C_2_Te_*x*_ maintains a high electrical conductivity (45 S m⁻^1^, 20 MPa), thereby ensuring efficient charge transport within the electrode. SEM images (Figs. 2b, c and S5) reveal the hallmark accordion-like morphology of MXene after Te etching, with uniformly expanded layers across the sample, indicating efficient and homogeneous etching. Notably, compared with samples from HF or Lewis-acidic melts, the Te-etched MXene exhibits more pronounced accordion-like morphology, implying enhanced interlayer separation with minimal damage to the M-X skeleton. Energy-dispersive spectroscopy (EDS) mapping (Fig. S6) confirms uniform Te distribution. Minor Al_2_Te_3_ and residual Te impurities were detected (Fig. S7), which was reduced after HCl washing, yielding to cleaner surface and more prominent MXene reflections. Element quantification analysis suggests a final composition of Ti_3_C_1.69_Te_2.93_O_0.64_. Figure 2d-f presents high-resolution X-ray photoelectron spectroscopy (XPS) spectra for Ti, C, and Te, unveiling chemical bonding states and compositions on the MXene surface. The Ti-C bond at ~ 454.92 eV (Ti 2*p*) and 281.97 eV (C 1*s*) remains prominent, demonstrating the preservation of the Ti_3_C_2_ framework without significant structural disruption. In the Te 3*d* spectra, the Te^2−^ signals at 572.95 eV (3*d*_5/2_) and 583.33 eV (3*d*_3/2_) corroborate the formation of Ti-Te bonding. The Te-Ti signal significantly outweighs any Te–O peak, indicating that in situ incorporation of Te leads to a higher fraction of Ti-Te bonds compared to previously reported two-step processes [34, 35]. The remaining elemental spectral information is shown in Fig. S8. This suggests reduced oxide formation, improved sample purity, and enhanced retention of the desirable Te-terminated structure. On the other hand, as shown in Fig. S9, a comparison of the samples stored under ambient air conditions for 9 months revealed that both the XRD patterns and microstructure remained nearly unchanged, indicating that the Te-functionalized MXene exhibits excellent environmental stability.

High-angle annular dark-field scanning transmission electron microscopy (HAADF-STEM) images and EDS line scans (Fig. 2g, h) further validate the successful etching of Al, with minimal residual content. Observing along the [11 $\overline{2 }$ 0] zone axis, Ti and Te atoms alternate along the c-axis, and the heavier Te atoms appear with enhanced contrast. A structural schematic (Fig. 2i) illustrates the spatial distribution of the elements, consistent with the measured interlayer spacing of ~ 12.9 Å. Theoretical diffraction analysis (Fig. 2a) indicates that Ti_3_C_2_Te_*x*_ exhibits a (002) reflection at 6.91° (d-spacing ~ 12.78 Å), closely matching our experimental observations. In contrast, Ti_3_C_2_Cl_2_ synthesized via Lewis-acidic melts shows the (002) peak at 7.96° (*d*-spacing ~ 11.09 Å) (Fig. S10). Hence, the Te-terminated MXene produced here possesses a notably larger interlayer spacing, which should be beneficial for electrochemical ion insertion and other applications [28]. Moreover, first-principles calculations reveal that Ti_3_C_2_Te_*x*_ has a work function and electronic structure indicative of high conductivity (Fig. 2j), exceeding that of halogen-functionalized MXene and only slightly below that of -OH-functionalized MXene. These results highlight the exceptional conductive properties of Te-terminated MXene.

Based on previously discussed Gibbs free energy calculations and our experimental findings, we propose the etching reaction to process by chemical equation (Eq. 1), which highlights the multifunctional role of Te in the etching mechanism and the formation of MXene materials:

1 $$2{\mathrm{Ti}}_{3} {\mathrm{AlC}}_{2} + \left( {3 + 2x} \right){\mathrm{Te}} = 2{\mathrm{Ti}}_{3} {\mathrm{C}}_{2} {\mathrm{Te}}_{x} + {\mathrm{Al}}_{2} {\mathrm{Te}}_{3}$$

### Generalization of the Te Etching Method

**Fig. 3:**
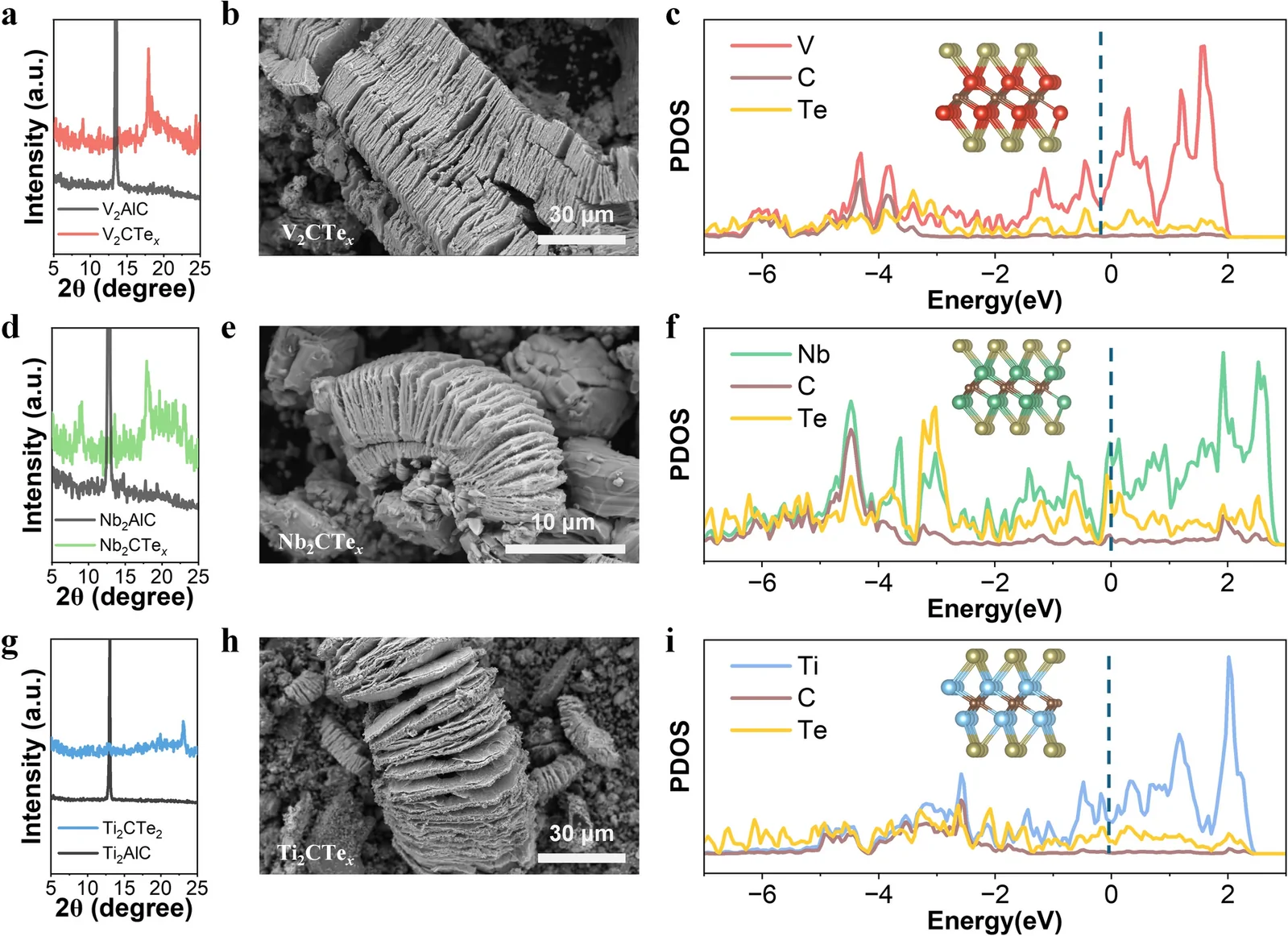
Preparation of various MXene. **a** XRD patterns of V_2_AlC and V_2_CTe_*x*_ MXene, **b** SEM image of V_2_CTe_*x*_ MXene before acid washing, and **c** theoretical calculation of optimized structure and projected density of states curve. **d** XRD patterns of Nb_2_AlC and Nb_2_CTe_*x*_ MXene, **e** SEM image of Nb_2_CTe_*x*_ MXene before acid washing, and **f** theoretical calculation of optimized structure and projected density of states curve. **g** XRD patterns of Ti_2_AlC and Ti_2_CTe_*x*_ MXene, **h** SEM image of Ti_2_CTe_*x*_ MXene before acid washing, and **i** theoretical calculation of optimized structure and projected density of states curve

Next, we investigated the generalization of the Te-based etching strategy using various MAX precursors, including V_2_AlC, Nb_2_AlC, and Ti_2_AlC. Under optimized conditions (Te/MAX = 3:1, 700 °C, Ar), selective removal of Al was confirmed in all cases (Figs. 3 and S12–S17), as evidenced by the disappearance of MAX diffraction peaks and the emergence of typical MXene reflections. The resulting powders displayed the characteristic accordion-like morphology. This approach is particularly advantageous for V- and Nb-based MAX systems, where conventional Lewis-acidic etching frequently yields low-quality MXene with a high level of impurities and severe oxidation (Fig. S11). In contrast, Te-based etching provides notably higher material quality and crystallinity. DFT calculations (Fig. 3c, f, i) confirm that V_2_CTe_*x*_, Nb_2_CTe_*x*_, and Ti_2_CTe_*x*_ are all structurally stable with Te groups adsorbed primarily atop the metal or carbon layers, enhancing structural integrity. The analysis of the calculated density of states suggests high electron density near the Fermi level, indicative of good electrical conductivity, a critical advantage for energy storage applications. These findings underscore the versatility of Te-based MXene, whose superior conductivity, stability, and tunable surface features could be exploited for a wide array of functional applications.

**Fig. 4:**
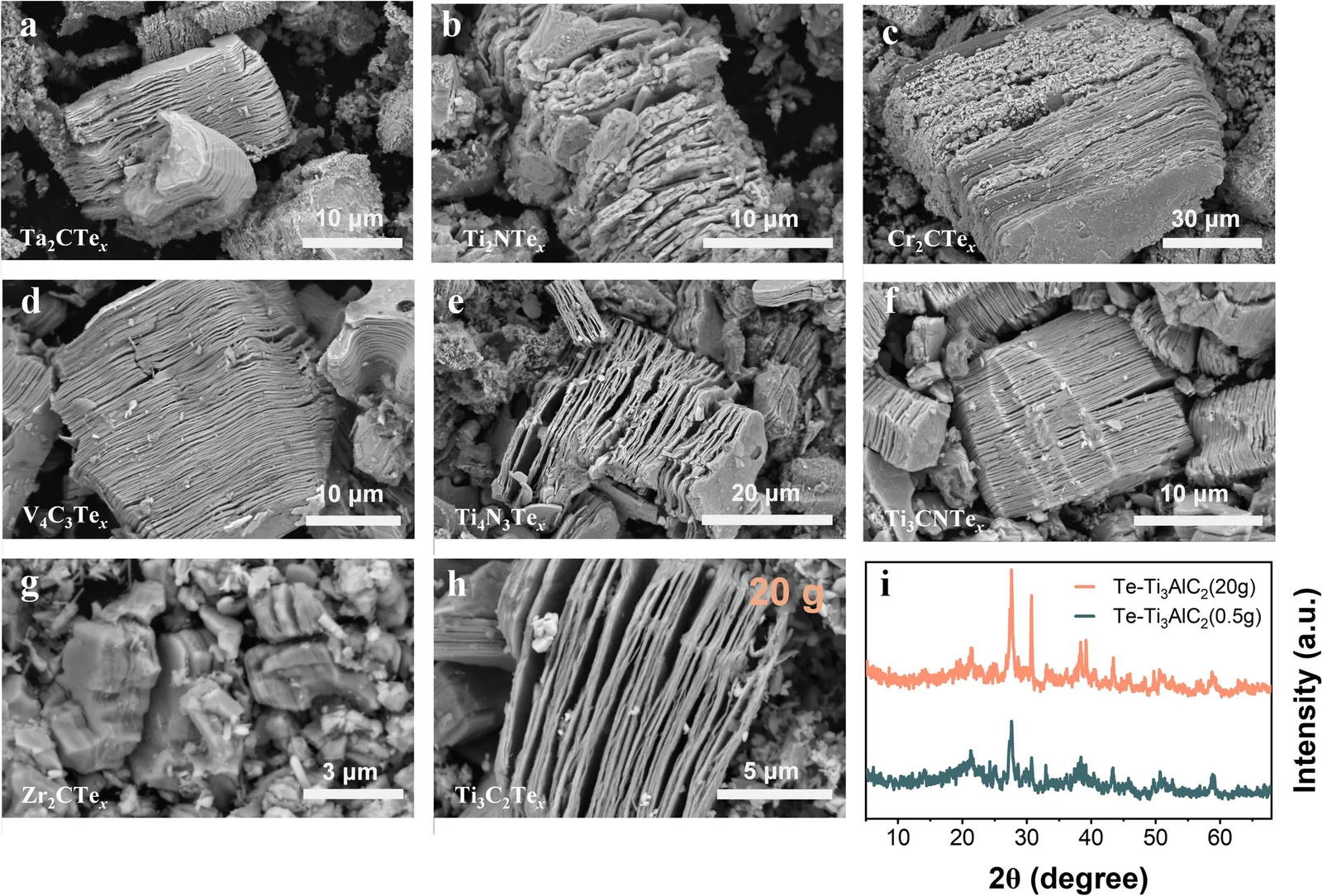
Universality and batch characterization. SEM images demonstrating the successful Te-based etching of various MAX phases, **a** Ta_2_CTe_*x*_. **b** Ti_2_NTe_*x*_. **c** Cr_2_CTe_*x*_. **d** V_4_C_3_Te_*x*_. **e** Ti_4_N_3_Te_*x*_. **f** Ti_3_CNTe_*x*_. **g** Zr_2_CTe_*x*_. Batch preparation of Ti_3_C_2_Te_*x*_ MXene. **h** SEM image and **i** XRD pattern of the product

We further broaden the scope of this synthetic route by extending it to the etching of 211-(Ti_2_AlN, Cr_2_AlC, Ta_2_AlC), 312-(Ti_3_AlCN), and 413-type (Ti_4_AlN_3_ and V_4_AlC_3_) of MAX phases. As shown in Figs. 4 and S18–S25, XRD and SEM data confirm effective etching across these diverse compositions, consistent with thermodynamic predictions that favor Al removal. Notably, only elemental Te and the MAX powder are required, both of which are stable under ambient conditions, which is an important practical advantage for large-scale manufacturing. More interestingly, upscaling the etching reaction to ~ 40-fold batches (20 g) resulted in comparable Te-terminated MXene quality as evidenced by SEM and XRD characterizations (Fig. 4h, i), confirming that this Te-based etching route is well suited for large-scale production. Furthermore, based on the preceding thermodynamic analysis, we explored the etching behavior of Se and S toward Ti_3_SiC_2_ (Fig. S26). The results show that Se effectively etched Ti_3_SiC_2_, while S failed to achieve etching due to its highly volatile nature during the process.

### Te-MXene Material for Sodium Storage

**Fig. 5:**
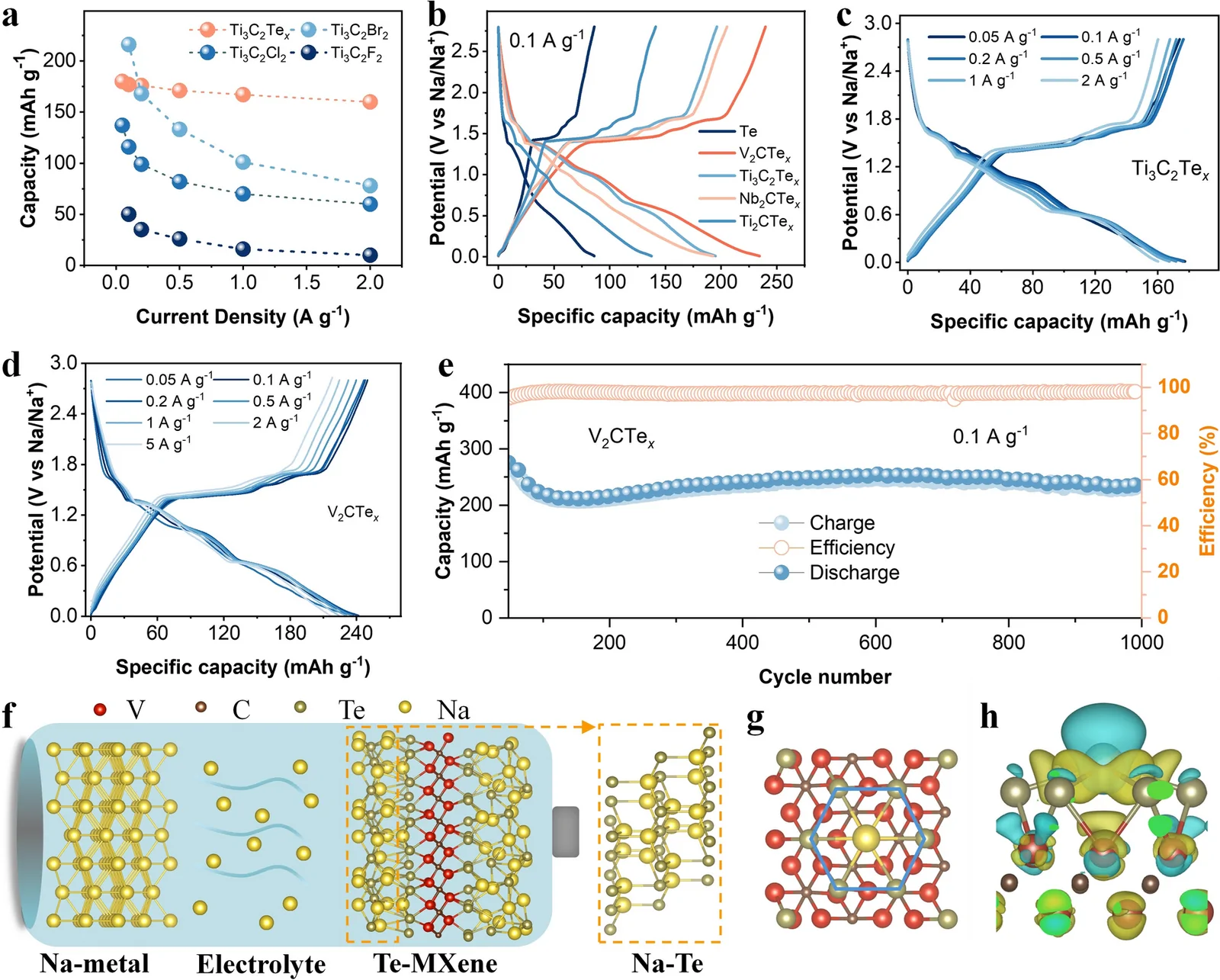
Sodium storage properties of Te-MXene.** a** Electrochemical performance comparison of sodium storage for Ti_3_C_2_Te_*x*_, Ti_3_C_2_Br_2_, Ti_3_C_2_Cl_2_ and Ti_3_C_2_F_2_. **b** Charge and discharge profiles of Te-based MXene electrodes at 0.1 A g^−1^. Rate-dependent charge and discharge curves of **c** Ti_3_C_2_Te_*x*_ and **d** V_2_CTe_*x*_. **e** Long-cycle stability of V_2_CTe_*x*_ at 0.1 A g^−1^. **f** Proposed storage mechanism for Te-based MXene materials. **g** Top-view depiction of optimized V_2_CTe_*x*_ MXene electrode material surface. **h** Differential charge distribution for Na adsorption at Te functional group vacancies on the surface of the V_2_CTe_*x*_ MXene electrode material

To elucidate how Te terminations influence electrochemical behavior, we achieved the electrochemical characterizations of Te-functionalized MXene during Na-ions intercalation [36]. Figure 5a compares the reversible charge storage capacity and rate performance of Ti_3_C_2_Te_*x*_, Ti_3_C_2_Br_2_, Ti_3_C_2_Cl_2_, and Ti_3_C_2_F_2_ at various current densities. At a current density of 0.05 A g^−1^, Ti_3_C_2_Cl_2_ delivers a capacity of 137 mAh g^−1^, while Ti_3_C_2_Te_*x*_ achieves a higher capacity of 180 mAh g^−1^. Moreover, Ti_3_C_2_Te_x_ retains 89% of its capacity (160 mAh g^−1^) at high specific current of 2 A g^−1^, whereas the Cl-terminated MXene shows only 44% capacity retention under the same conditions. In contrast, the overall electrochemical performance of Ti_3_C_2_Br_2_ and Ti_3_C_2_F_2_ is poor. The lithium-ion storage performance of Te-functionalized MXene was evaluated. As shown in Fig. S27, both types of Te-terminated MXene materials exhibit rapid capacity fading and poor cycling stability when used as electrodes in lithium-ion batteries. This inferior performance is likely due to the absence of the Na-Te alloying mechanism that plays a key role in enhancing capacity and cycling stability in sodium-ion systems. The observations highlight that Te functionalities not only increase the storage capacity but also markedly enhance the rate capability of MXene electrodes in sodium-ion batteries. We further investigated the electrochemical behavior of four different Te-functionalized MXene (Ti_3_C_2_Te_*x*_, V_2_CTe_*x*_, Ti_2_CTe_*x*_, and Nb_2_CTe_*x*_), compared with the elemental Te (Fig. 5b). Their charge–discharge profiles show a distinctive plateau around 1.5 V vs. Na^+^/Na commonly associated with Na-Te alloying, which is corroborated by differential capacity (dQ/dV) curves (Fig. S28). Among these, V_2_CTe_*x*_ delivers the highest capacity of 247 mAh g^−1^. Figure 5c, d depicts typical charge–discharge curves at various rates for Ti_3_C_2_Te_*x*_ and V_2_CTe_*x*_, respectively, demonstrating excellent rate performance with minimal polarization. Even at 5 A g^−1^ (corresponding to 23 C-rate), V_2_CTe_*x*_ retains 216 mAh g^−1^. Long cycling of V_2_CTe_*x*_ at 0.1 A g^−1^ reveals capacities above 235 mAh g^−1^ after 1000 cycles, underscoring outstanding cyclic stability. Even under high current cycling at 2 A g^−1^ (Fig. S29), the V_2_CTe_*x*_ electrode exhibited excellent cycling stability. Compared to conventional hard carbon materials, the Te-functionalized MXene exhibits superior rate capability and a higher voltage plateau, which not only contributes to higher power density but also helps effectively avoid dendrite formation during cycling. Previous studies have shown that tellurium undergoes a Na-Te alloying process in sodium-ion batteries [37, 38]. However, Te-based negative electrodes often suffer from large volumetric changes during alloying/dealloying, leading to structural instability. In addition, we conducted sodium storage tests on pure Te electrodes within this system. As shown in Fig. S30, the overall capacity was relatively low and exhibited continuous fading during cycling. Here, the surface-bound nature of Te on 2D MXene scaffolds is believed to provide a promising solution (Figs. 5g and S31a, b), in which the alloying reaction may predominantly involve Te groups that partially detach from the MXene surface to form Na-Te [38], thereby helping to preserve the layered MXene framework and reduce mechanical degradation. As shown in Fig. S32, post-cycling disassembly of the V_2_CTe_*x*_-based cell revealed that the primary species deposited on the separator was Na-Te alloy, which is consistent with the previous analytical results. DFT calculations (Figs. 5h and S31c, d) further indicate that Na ions react preferentially with surface Te groups, leaving behind vacancies on the MXene surface, which can subsequently absorb additional Na. The post-cycling SEM observations revealed that the V_2_CTe_*x*_ electrode still retained a well-defined layered morphology, indicating good structural stability during long-term cycling. X-ray photoelectron spectroscopy (XPS) analysis was conducted on the V_2_CTe_*x*_ electrode after cycling in the discharged state. The Te spectrum is presented in Fig. S33. Compared with the pristine V_2_CTe_*x*_, the Te functional group content significantly decreased, as indicated by the marked reduction in Te peak intensity. This is attributed to the partial desorption or transformation of Te functional groups during electrochemical cycling, resulting in a reduced surface content. Moreover, a distinct Te-Na bonding peak appeared at approximately 572 eV, further confirming that after the detachment of Te functional groups, the resulting Te vacancies can effectively facilitate Na⁺ adsorption and bonding. These findings are in good agreement with the results of the ex-situ XRD analysis and the analysis of products on the separator surface. These insights underscore that Te-based MXene uniquely integrates the advantages of 2D layered architectures with the high capacity contributions of chalcogenide alloying, thereby delivering exceptional performance and stability in sodium-ion storage. The assembled full cell exhibited a specific capacity of 104 mAh g^−1^ at 0.1 A g^−1^ (Fig. S34), with a voltage profile positioned between the average voltages of the cathode and anode in their respective half-cells. Collectively, these electrochemical results highlight the significant potential of Te-based MXene materials for high-performance sodium-ion batteries and further advance the practical deployment of MXene-based materials in next-generation energy storage systems.

## Conclusion

In summary, we present a controllable synthesis strategy for MXene based on elemental Te, which leverages the redox reactivity of Te to achieve simultaneous selective etching of Al from the MAX phase and in situ Te functionalization. Compared with conventional methods, this one-step approach obviates additional surface functionalization steps and markedly simplifies synthesis. Our results demonstrate the broad applicability and scalability of the Te-based route across multiple MAX systems, consistently yielding Te-terminated MXene with well-defined “accordion-like” morphologies. The introduction of Te functional groups endows MXene with enhanced structural stability, larger interlayer spacing, and improved electrical conductivity, leading to substantially boosted electrochemical sodium-ion storage performance. V_2_CTe_*x*_ achieves 247 mAh g^−1^ at 0.05 A g^−1^ and retains 216 mAh g^−1^ even at 2 A g^−1^ (23 C). Our findings indicate that elemental etching, exemplified here by Te, offers a new paradigm for tuning MXene surface chemistry and optimizing device-level performance, underscoring the fundamental importance and far-reaching potential of MXene in energy storage applications.

## Supplementary Information

Below is the link to the electronic supplementary material.

Supplementary file1 (DOCX 14,852 KB)
