# Supplementary material for: Tellurium-Terminated MXene Synthesis via One-Step Tellurium Etching
Source: Nanomicro Lett. 2025 Aug 11;18:28. doi: 10.1007/s40820-025-01875-1 (PMC12339786; doi:10.1007/s40820-025-01875-1)
Supplement: Supplementary file 1 — Supplementary file1 (DOCX 14,852 KB) [file 40820_2025_1875_MOESM1_ESM.docx]

Supporting Information for

**Tellurium Terminated MXene Synthesis via One-Step Tellurium Etching**

Guoliang Ma^1^, Zongbin Luo^1^, Hui Shao^2^*, Yanbin Shen^2^, Zifeng Lin^1^*, Patrice Simon^3*^

^1^College of Materials Science and Engineering, Sichuan University, Chengdu 610065, P. R. China

^2^ i-Lab, CAS Center for Excellence in Nanoscience, Suzhou Institute of Nano-Tech and Nano-Bionics (SINANO), Chinese Academy of Sciences (CAS), Suzhou 215123, P. R. China

^3^CIRIMAT, Université de Toulouse, CNRS, France

*Corresponding authors. E-mail: [simon@chimie.ups-tlse.fr](mailto:simon@chimie.ups-tlse.fr) (Patrice Simon); [linzifeng@scu.edu.cn](mailto:linzifeng@scu.edu.cn) (Zifeng Lin); [hshao2023@sinano.ac.cn](mailto:hshao2023@sinano.ac.cn) (Hui Shao)

**S1 Materials**

Ti_3_AlC_2_, V_2_AlC, Ti_2_AlC, Nb_2_AlC, Ta_2_AlC, Ti_2_AlN, Ti_3_AlCN, Ti_4_AlN_3_, V_4_AlC_3_, Cr_2_AlC, Zr_2_AlC etc. MAX (99% purity, 400 mesh-600 mesh) was purchased from Jilin Yiyi Technology Co. Ltd, Te (99.99% purity, 300 mesh) was purchased from Hebei Jincan Metals New Material Co. Ltd., HCl solution was purchased from Chengdu Kelong Company, 1 m NaPF_6_ in ethylene glycol dimethyl ether (DME, battery-grade) electrolyte was purchased from DuoDuo ElectroChemical Reagent, and glass fibers (GF/D and GF/A) was purchased from Kelude Company, all the chemicals and reagents were used directly after purchase.

**S2 Synthesis of Te-MXene**

Taking the synthesis of Ti_3_C_2_Te*_x_* MXene as an example, the precursor materials, Ti_3_AlC_2_ (0.5 g) and Te powder (0.975 g), are weighed in a stoichiometric ratio of 1:3. A small amount of ethanol is added to facilitate mixing, and the mixture is manually homogenized until ethanol evaporates. The mixture is then transferred to a quartz boat and placed in a tube furnace. To ensure an inert Ar atmosphere, the system is first flushed with argon gas at a flow rate of 600 mL min^-1^ for 10 minutes to expel any residual air from the quartz tube. The flow rate is subsequently adjusted to 100 mL min^-1^ to maintain the inert environment. The furnace is then heated to 700°C at a rate of 10°C min^-1^ and held at this temperature for 1 hour before being cooled to room temperature. Post-treatment involves washing the product with 100 mL of 1 m HCl solution for 1 hour to remove residual impurities. This is followed by multiple washes with deionized water using a filtration system to eliminate any floating impurities on the surface. Finally, the material is vacuum-dried at 60°C for 8–12 hours to obtain the Ti_3_C_2_Te*_x_* MXene. The preparation of other MXene materials follows a similar procedure, with variations in temperature and precursor ratios depending on the specific MAX phase. Detailed information on these variations is provided in Table S7 of the Supporting Information.

**S3 Material characterization**

The phase composition of the MXene products was analyzed using X-ray diffraction (XRD) with a Cu target on a DX-2700 diffractometer. The scanning range was 5°–70°, with an operating tube voltage of 40 kV and a tube current of 30 mA. The microstructure and surface elemental composition of the products were examined using a JEOL field emission scanning electron microscope (FE-SEM, JSM-7900F) equipped with an energy-dispersive X-ray spectrometer (EDS, UltimMax65). Focused ion beam (FIB) sample preparation for particle cross-sections was performed using an FEI HELIOS NanoLab 600i system. Atomic-level analysis of the products was carried out using an FEI Titan Themis 80-300 spherical aberration-corrected transmission electron microscope, which is equipped with two aberration correctors. The elemental valence states of the MXene products were analyzed via X-ray photoelectron spectroscopy (XPS) using an AXIS Supra system (Kratos) with a monochromatic Al Kα X-ray source. The binding energy data were calibrated to the C 1s peak at 284.6 eV. The contents of Ti and Te elements were quantified using an ICAP 7000 series inductively coupled plasma atomic emission spectrometer (ICP-AES). The C content was determined with a CS600 carbon and sulfur analyzer, while the oxygen content of the MXene materials was measured using a TCH600 oxygen, nitrogen, and hydrogen analyzer.

**S4 Theoretical calculation**

The theoretical calculations based on Density Functional Theory (DFT) were performed using the DS-PAW software package integrated within Device Studio [S1]. For the pseudopotential, the exchange-correlation functional was treated using the Perdew-Burke-Ernzerhof generalized gradient approximation (GGA-PBE). A vacuum layer of 20 Å was added in the structure to minimize periodicity effects along the c-axis. Geometry optimizations were carried out using a 5x5x1 Monhkorst-Pack k-point grid, while electronic structure calculations employed a 7x7x1 grid. The convergence criterion for forces was set to 0.02 eV Å^-1^, with a plane-wave energy cutoff of 600 eV and an energy convergence threshold of less than 10^-6^ eV. Long-range van der Waals interactions were accounted for using the DFT-D3 method with Becke-Jonson damping corrections.

Gibbs Free Energy Calculation: The Gibbs free energy (ΔG_r_) was calculated using the HSC Chemistry software (version 9.5), with the following reaction Equation S1:

$\Delta G_{r}=\Delta H_{r}+T\Delta S_{r}$ (S1)

$\Delta H_{r}$ represents the reaction enthalpy change, $\Delta S_{r}$ represents the entropy change, T represents the reaction temperature. Additionally, $\Delta H_{f}$and $\Delta S_{f}$values can be obtained from HSC thermodynamic software. Based on prior research experience, the entire preparation process can be viewed as a dynamic equilibrium at constant pressure. Furthermore, to predict the etching effect of Te on MAX precursors, we selected elements A from MAX and Te as the initial reactants [S2, S3]. The etching process equations are as follows:

$2A+3Te={Al}_{2}{Te}_{3}$ (S2)

Here, the A elements mainly include Al, In, and Ga (Equation S2).

$A+Te=ATe$ (S3)

In this case, the A elements primarily include Si, Zn, Sn, Ge, and Pb (Eq. S2).

The details are listed in Tables S1 and S2.

**S5 Supplementary Figures and Tables**

**Table S1** Gibbs free energy (G_r_) for the reaction of Te with A-site elements in MAX

| **A-site** | **Te** | **S** | **Se** | **P** | **Sb** | **As** |
| --- | --- | --- | --- | --- | --- | --- |
| **Al** | -279.96 | 73.63 | 79.29 | -85.02 | -37.88 | -109.61 |
| **Si** | 60.02 | -201.09 | -118.40 | -35.52 | None | None |
| **Ga** | -206.05 | -174.10 | -125.44 | -68.68 | -16.51 | -44.42 |
| **In** | -135.79 | -102.67 | -63.68 | -34.14 | -17.92 | -35.02 |
| **Zn** | -99.20 | -177.44 | -70.40 | -2.91 | -15.41 | -6.98 |
| **Pb** | -55.147 | -84.71 | -87.00 | None | None | None |
| **Sn** | -49.43 | -92.52 | -69.61 | None | None | None |
| **Ge** | -52.40 | -104.44 | -98.02 | 39.97 | None | -83.09 |


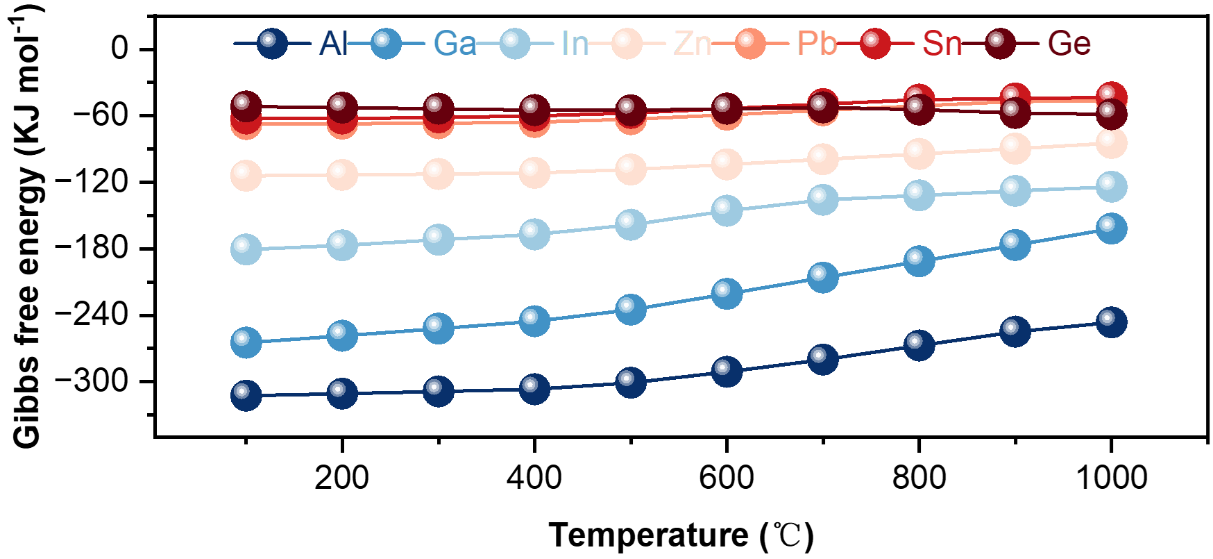


**Fig. S1** The Gibbs free energy of the reaction between different A- site elements (Al, Ga, In, Zn, Pb, Sn, Ge) and Te at different temperatures

**Table S2** Gibbs free energy (Gr) for the reaction of Te with A-site elements in different MAX at different temperatures

| **Gibbs Free Energy (∆G) of different A-site elements (kJ mol^-1^)** | | | | | | | | | | |
| --- | --- | --- | --- | --- | --- | --- | --- | --- | --- | --- |
| **A** | **100 ℃** | **200 ℃** | **300 ℃** | **400 ℃** | **500 ℃** | **600 ℃** | **700 ℃** | **800 ℃** | **900 ℃** | **1000 ℃** |
| **Al** | -312.630 | -310.806 | -308.866 | -306.830 | -300.965 | -291.049 | -279.963 | -267.317 | -254.984 | -246.552 |
| **Zn** | -7.979 | -8.287 | -8.633 | -9.130 | -45.479 | -46.760 | -47.605 | -48.281 | -48.818 | -49.240 |
| **Sn** | -62.435 | -62.508 | -61.550 | -60.115 | -57.435 | -53.448 | -49.430 | -45.401 | -44.302 | -43.410 |
| **In** | -180.654 | -176.793 | -171.923 | -167.003 | -158.356 | -145.805 | -135.794 | -131.836 | -127.901 | -123.988 |
| **Ga** | -264.734 | -258.530 | -252.086 | -245.492 | -235.071 | -220.646 | -206.051 | -191.351 | -176.588 | -161.798 |
| **Ge** | -51.699 | -52.653 | -53.715 | -54.927 | -54.993 | -53.725 | -52.405 | -54.453 | -57.408 | -58.640 |
| **Pb** | -67.478 | -67.105 | -66.678 | -65.619 | -63.048 | -59.130 | -55.147 | -51.124 | -47.077 | -46.670 |


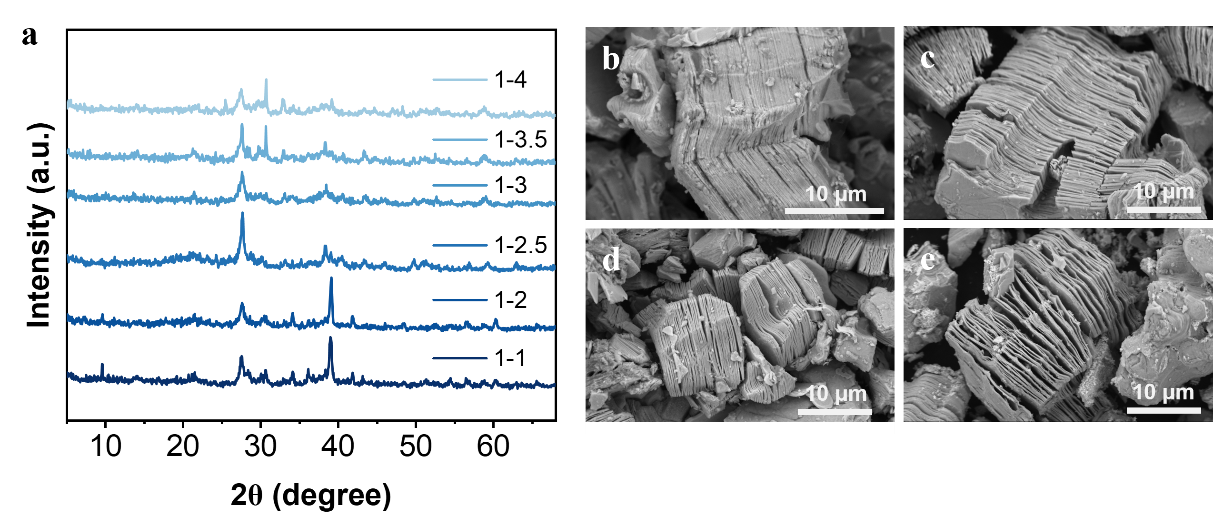


**Fig. S2** **a** Ti_3_C_2_Te*_x_* different ratios XRD, different ratios SEM **b** 1-2, **c** 1-2.5, **d** 1-3, **e** 1-3.5


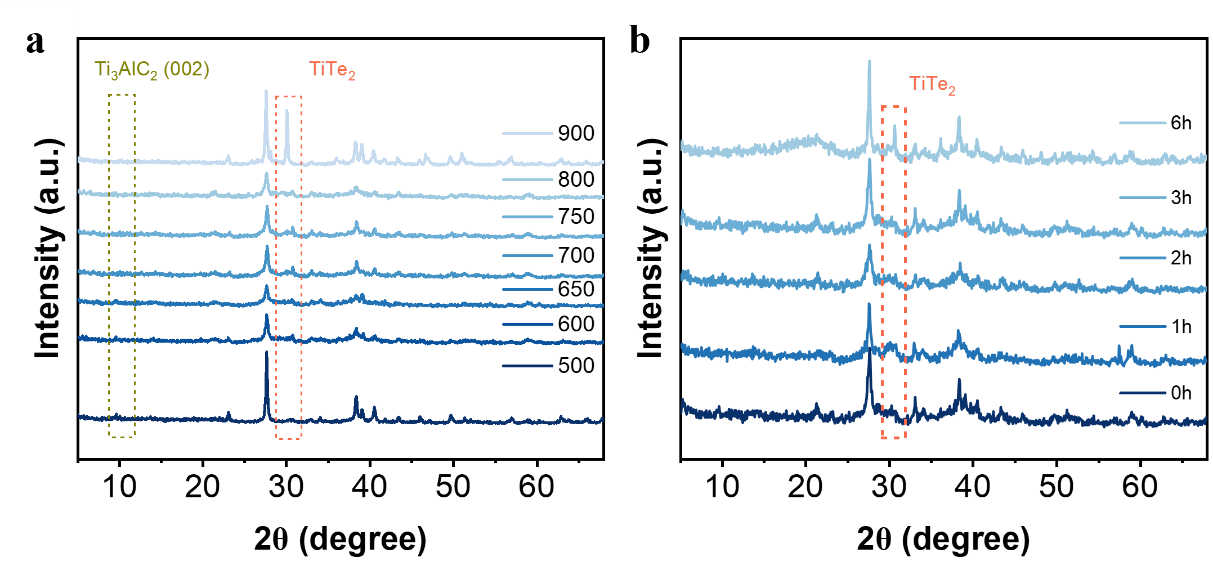


**Fig. S3 a** Ti_3_C_2_Te*_x_* XRD at different temperatures **b** XRD patterns of Ti_3_C_2_Te*_x_* synthesized at 700 °C during different reaction times


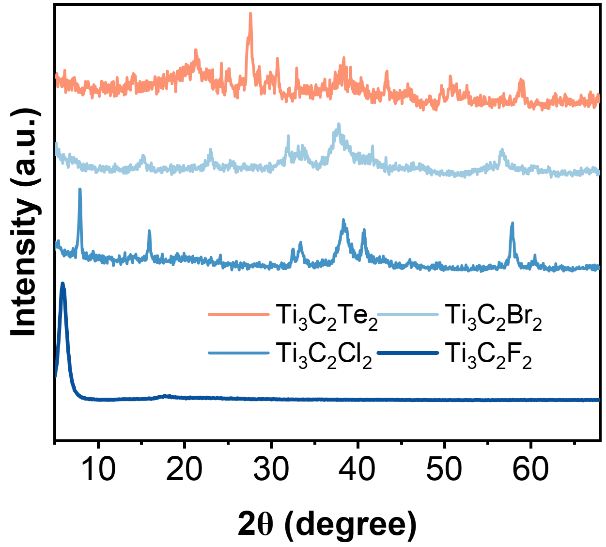


**Fig. S4** The XRD of -F, -Cl, -Br, -Te functional groups Ti_3_C_2_ MXene


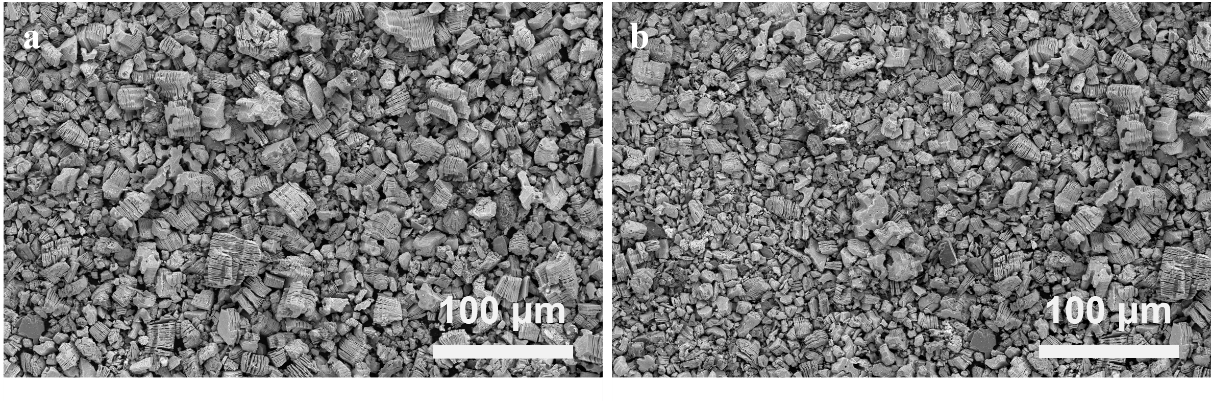


**Fig. S5** Ti_3_C_2_Te*_x_* SEM microstructure **a** 300x, **b** 500x


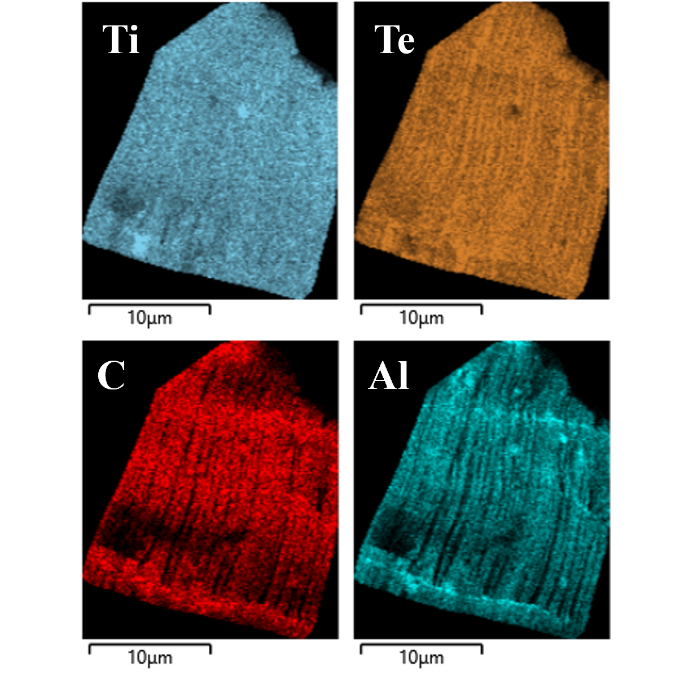


**Fig. S6** Ti_3_C_2_Te*_x_* EDS surface element distribution data


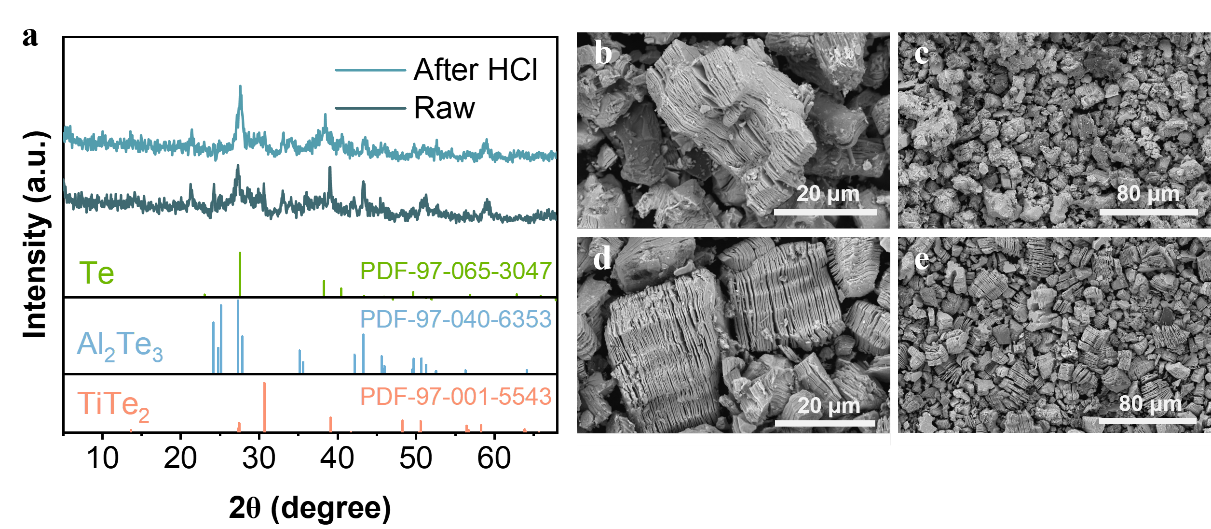


**Fig. S7** Ti_3_C_2_Te*_x_* products before and after HCl post-treatment. **a** XRD, **b-c** SEM of the product before acid washing, **d-e** SEM of the product after acid washing

**Table S3** Composition of the Ti_3_C_2_Te*_x_* MXene

| **Element** | **wt.%** | **mol%** |
| --- | --- | --- |
| Ti | 25.81 | 3 |
| C | 3.63 | 1.69 |
| Te | 67.13 | 2.93 |
| O | 1.85 | 0.64 |
| **Composition** | Ti_3_C_1.69_Te_2.93_O_0.64_ | |


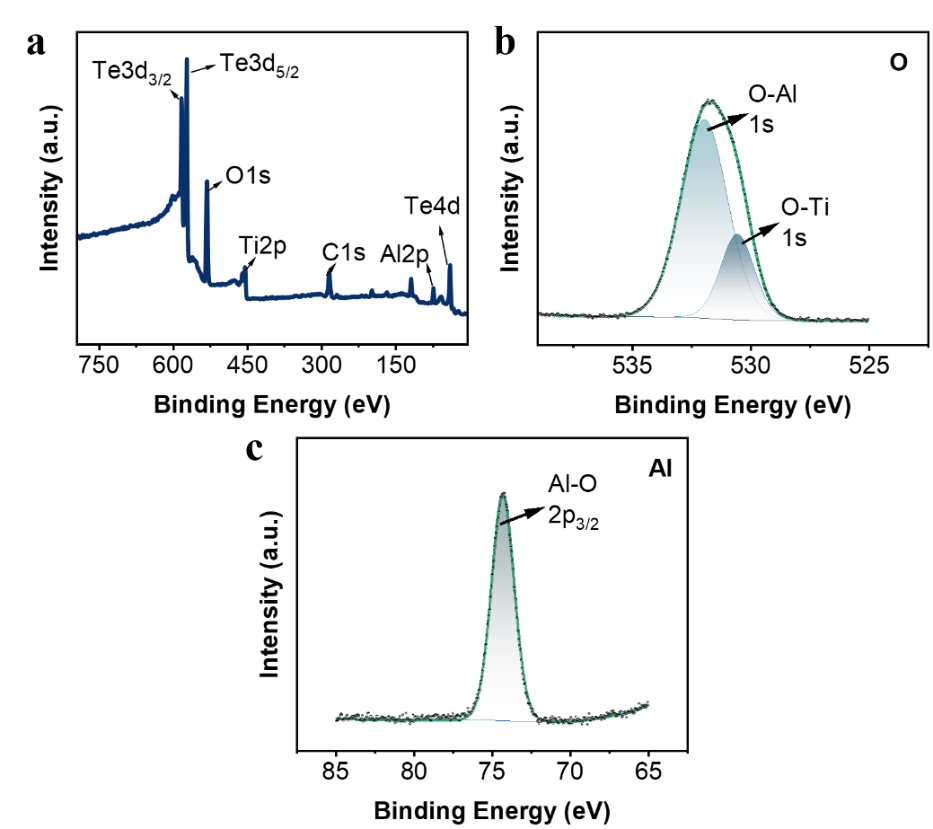


**Fig. S8** Ti_3_C_2_Te*_x_* XPS data **a** total spectrum, **b** O-fraction, **c** Al-fraction

**Table S4** XPS Peak position information of Ti_3_C_2_Te*_x_*

| **Region** | **BE(eV)** | **FWHM(eV)** | **Assigned** | **Reference** |
| --- | --- | --- | --- | --- |
| Ti 2p_3/2_(2p_1/2_) | 454.92(460.97) | 0.92(0.96) | Ti-C | [4, 5] |
|  | 455.82(461.99) | 2.37(1.02) | Ti-Te | [S4, S5] |
|  | 459.16(464.6) | 1.75(2.69) | Ti-O | [S4, S5] |
| C 1s | 281.97 | 0.92 | C-Ti | [S4, S5] |
|  | 284.47 | 1.38 | C-C | [S4, S5] |
|  | 285.85 | 2.06 | C-H | [S4, S5] |
|  | 288.67 | 2.03 | -COO | [S4, S5] |
| Te 3d | 572.95(583.33) | 1.24(1.18) | Te-Ti | [S4, S5] |
|  | 572.91(583.27) | 2.84(2.84) | Te | [S4, S5] |
|  | 576.21(587.16) | 2.49(2.49) | Te^δ+^ | [S4, S5] |
| O 1s | 530.56 | 2.33 | O-Ti | [S4, S5] |
|  | 531.96 | 1.69 | O-Al | [S4, S5] |


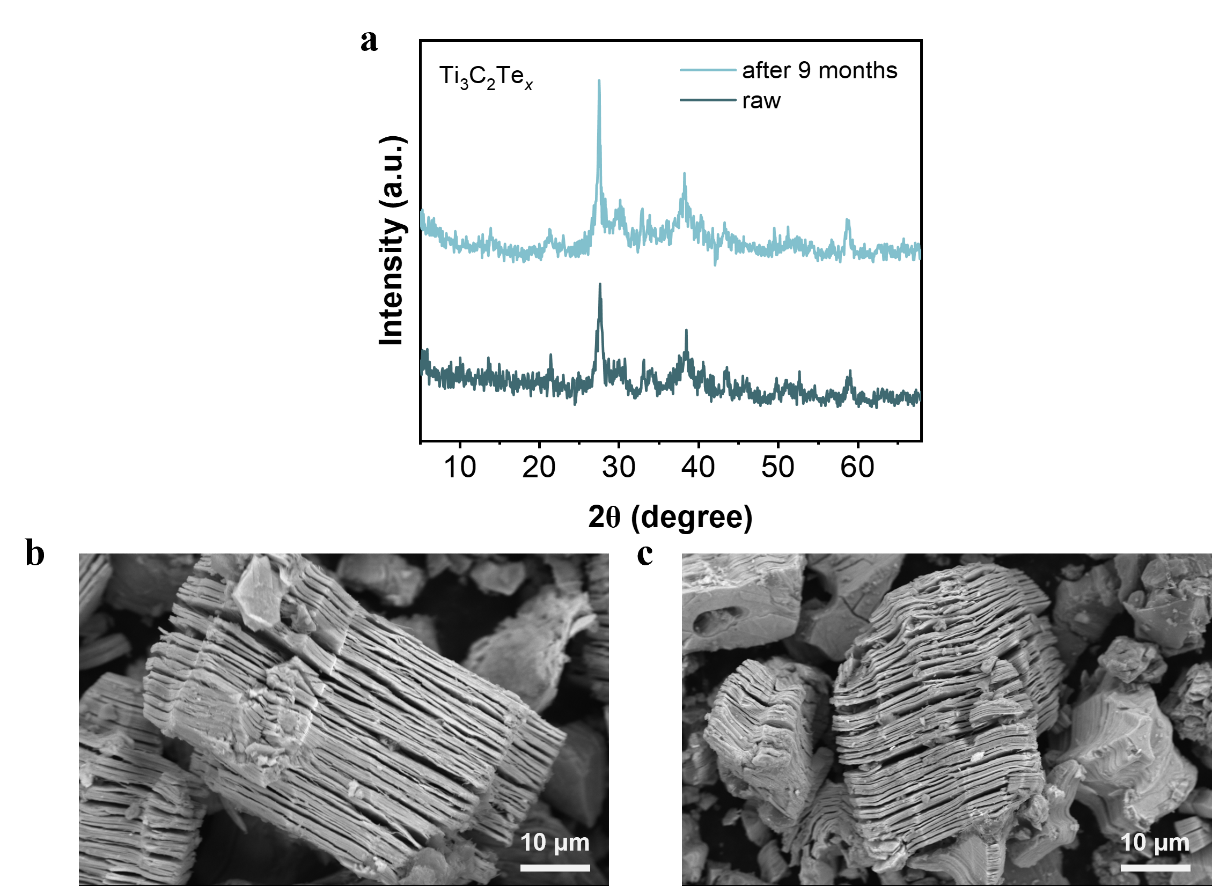


**Fig. S9** Ti_3_C_2_Te*_x_* products after 9 months in room temperature air environment. **a** XRD, **b** SEM of the product, **c** SEM of the product after 9 months


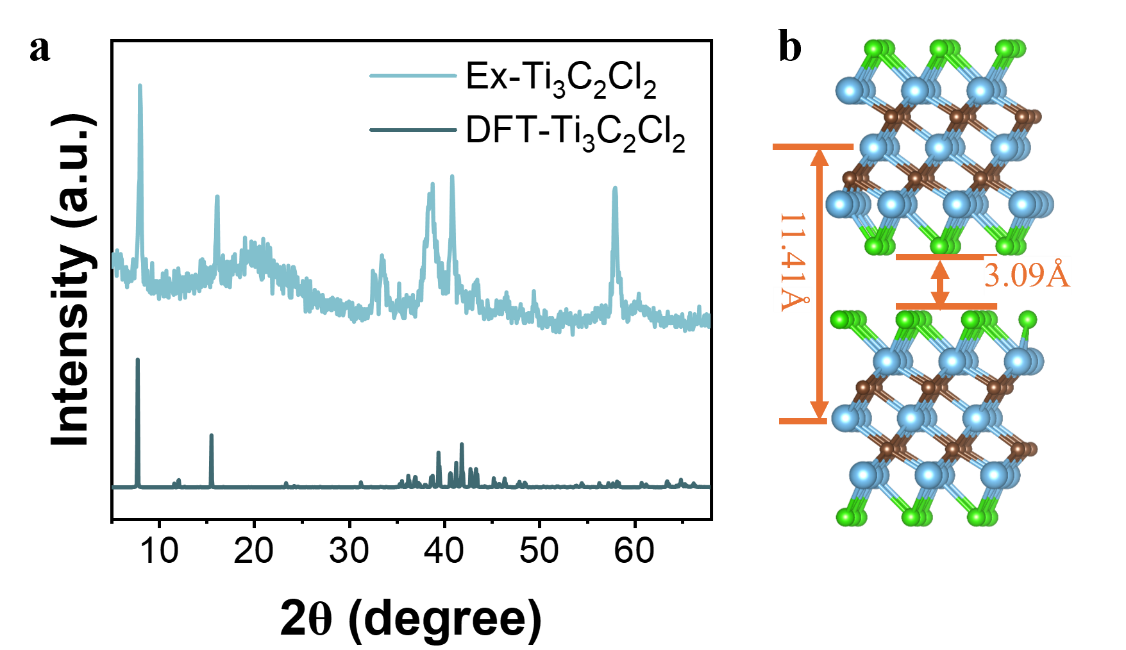


**Fig. S10** **a** Experimental and theoretically calculated XRD of Ti_3_C_2_Cl_2_, **b** Theoretical structure of Ti_3_C_2_Cl_2_


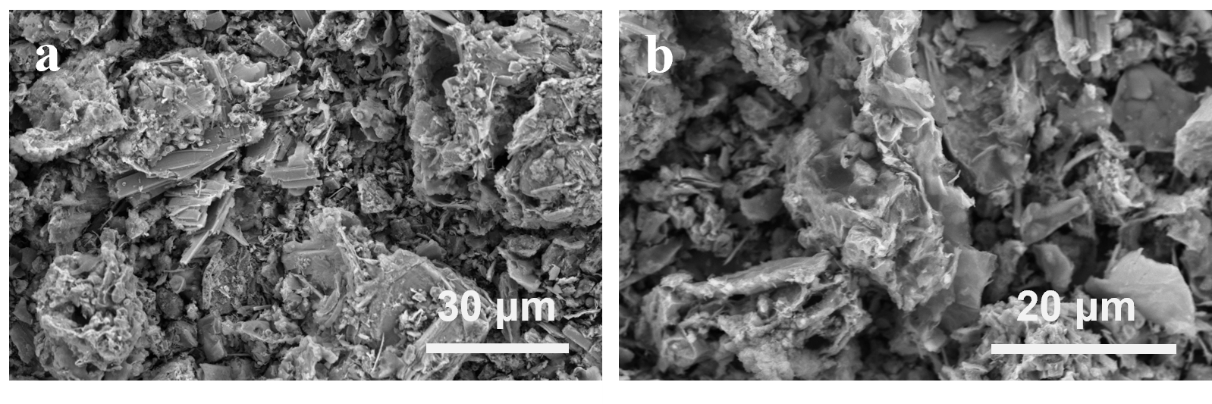


**Fig. S11** Lewis acid salt CuCl_2_ etching of V_2_AlC. **a-b** SEM microstructure


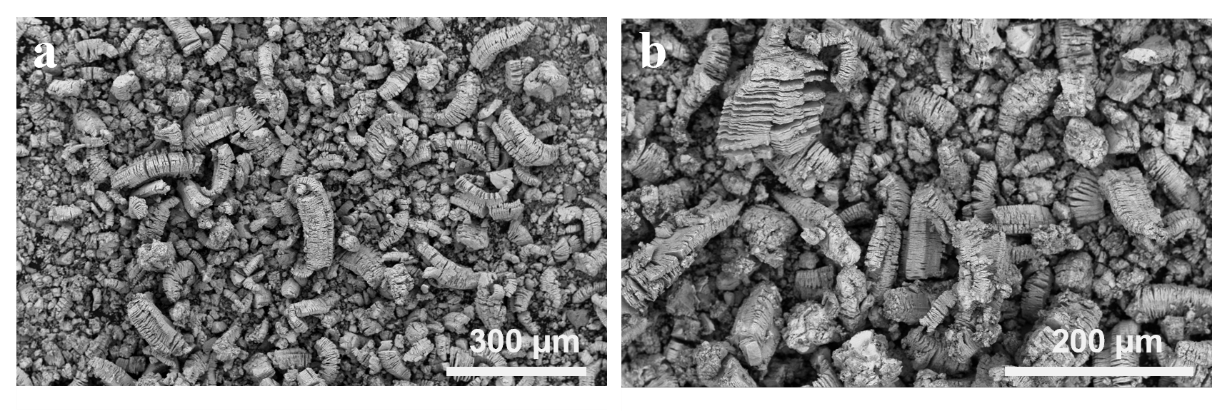


**Fig. S12** Microscopic morphology of V_2_CTe*_x_* MXene without post-treatment SEM, **a** 1000x, **b** 200x


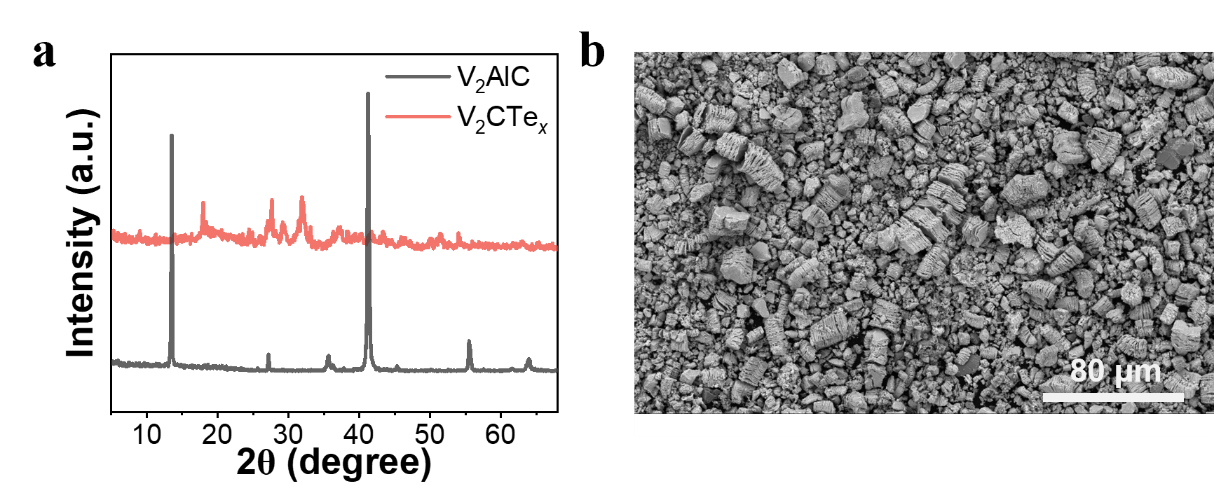


**Fig. S 13** V_2_CTe*_x_* MXene. **a** XRD before and after etching. **b** SEM macrostructure after acid cleaning


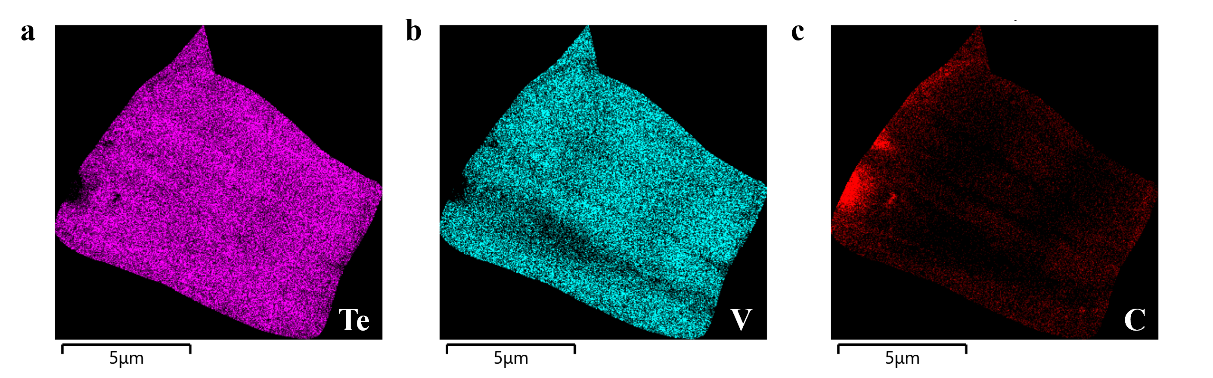


**Fig. S14** V_2_CTe*_x_* MXene EDS data **a** Te. **b** V. **c** C

**Table S5** Composition of the V_2_CTe*_x_* MXene

| **Element** | **wt%** | **mol%** |
| --- | --- | --- |
| V | 26.78 | 2 |
| C | 2.7 | 0.85 |
| Te | 67.1 | 2 |
| O | 1.33 | 0.31 |
| **Composition** | V_2_C_0.85_Te_2_O_1.71_ | |


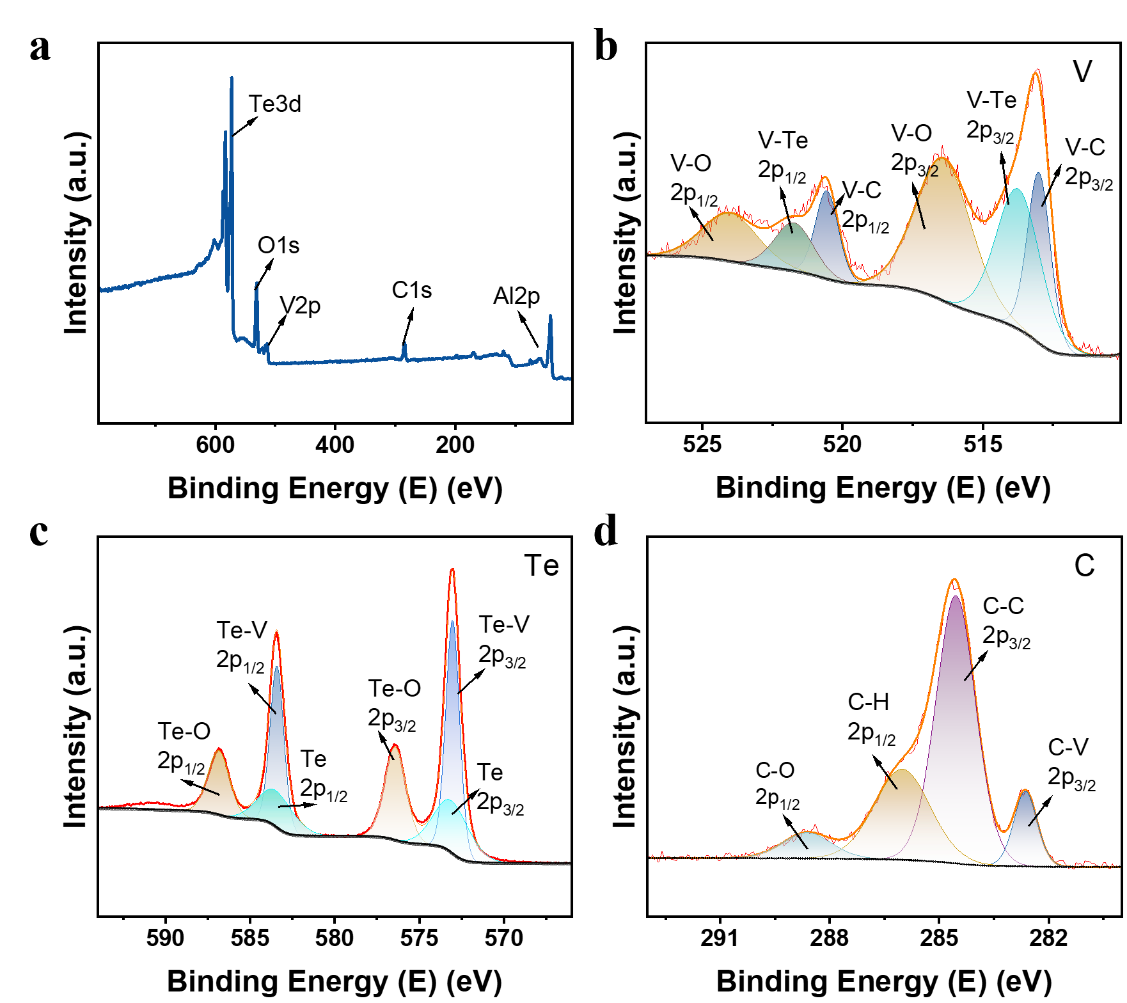


**Fig. S15** V_2_CTe*_x_* XPS data **a** total spectrum, **b** V-fraction, **c** Te-fraction, **d** C-fraction

**Table S6** XPS Peak position information of V_2_CTe*_x_*

| **Region** | **BE(eV)** | **FWHM(eV)** | **Assigned** | **References** |
| --- | --- | --- | --- | --- |
| V 2p_3/2_(2p_1/2_) | 513.0(520.56) | 1.01(1.01) | V-C | [S4, S5] |
|  | 513.74(521.74) | 1.94(1.71) | V-Te | [S4, S5] |
|  | 516.4(524.01) | 2.44(2.42) | V-O | [S4, S5] |
| C 1s | 282.63 | 0.79 | C-Ti | [S4, S5] |
|  | 284. 54 | 1.34 | C-C | [S4, S5] |
|  | 286.01 | 1.87 | C-H | [S4, S5] |
|  | 288.62 | 1.65 | -COO | [S4, S5] |
| Te 3d | 572.79(583.19) | 1.24(1.24) | Te^2-^ | [S4, S5] |
|  | 572.90(583.05) | 3.26(3.26) | Te^δ+^ | [S4, S5] |
|  | 576.13(587.08) | 2.72(2.72) | Te | [S4, S5] |
| O 1s | 530.7 | 1.56 | O-V | [S4, S5] |
|  | 533.0 | 2.06 | O-Al | [S4, S5] |


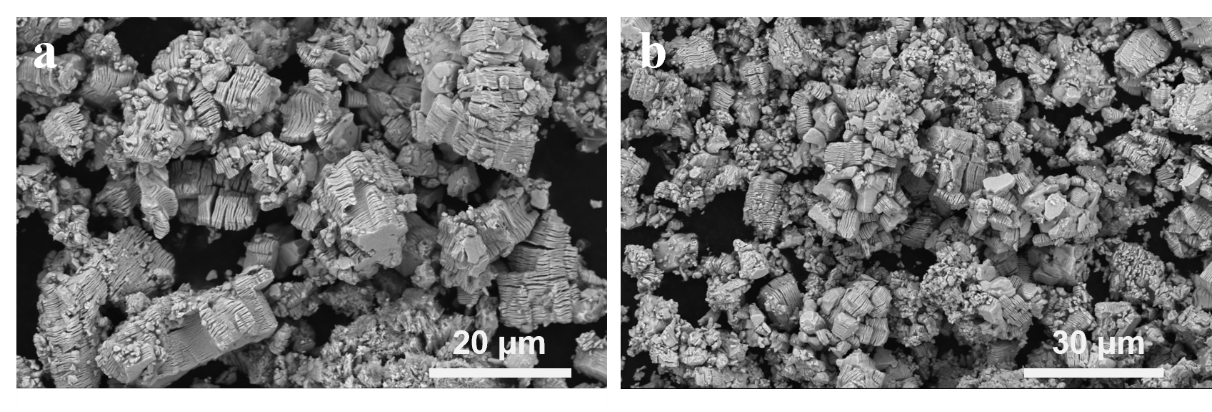


**Fig. S16** Microscopic morphology of Nb_2_CTe*_x_* MXene without post-treatment SEM. **a** 4000x. **b**1000


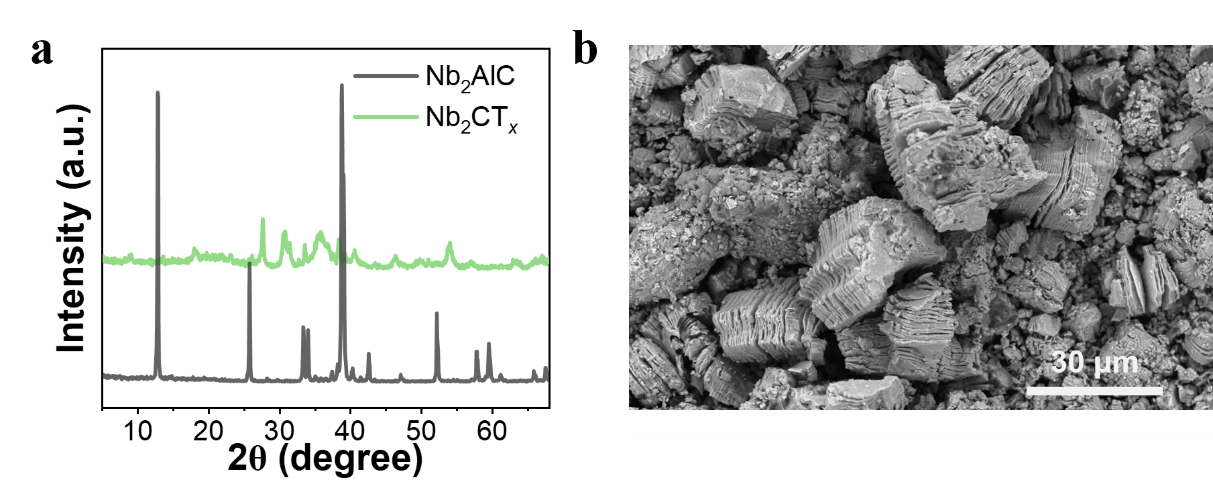


**Fig. S17** Nb_2_CTe*_x_* MXene. **a** XRD before and after etching. **b** SEM macrostructure after acid cleaning


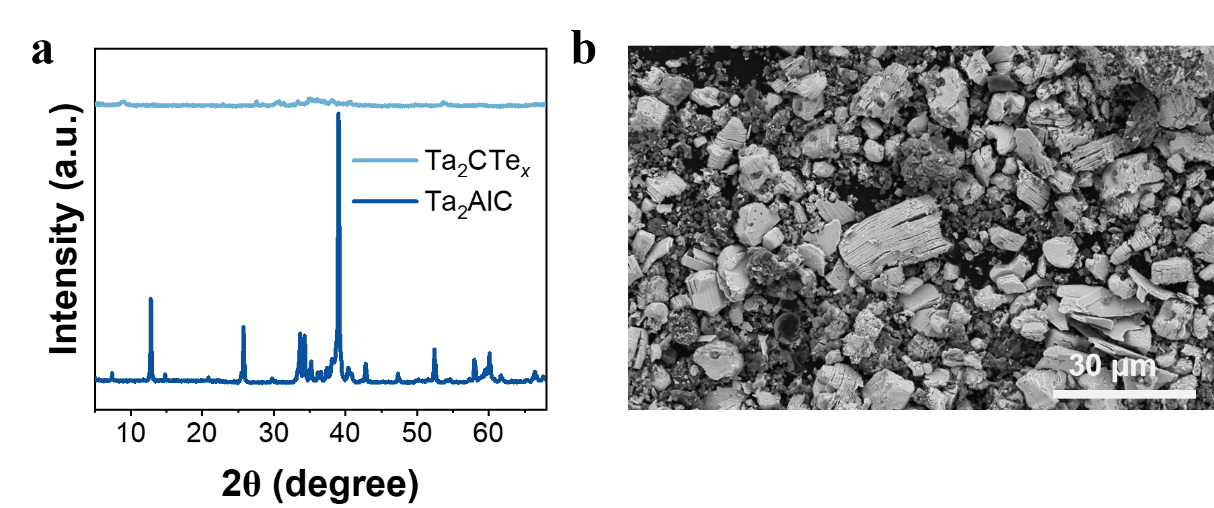


**Fig. S18** Ta_2_CTe*_x_* MXene. **a** XRD before and after etching. **b** SEM macrostructure after acid cleaning 1000x


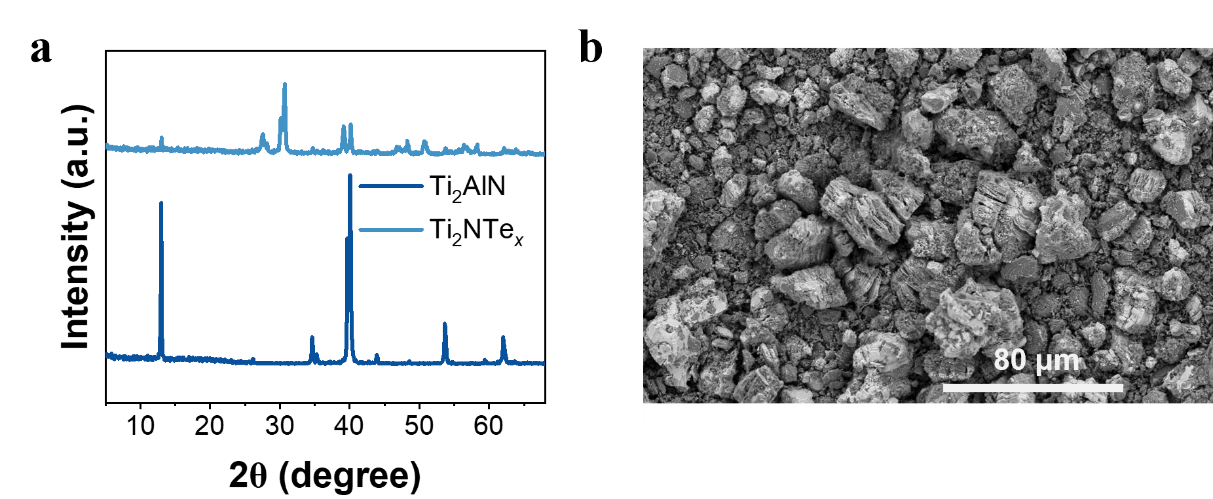


**Fig. S19** Ti_2_NTe*_x_* MXene. **a** XRD before and after etching. **b** SEM macrostructure after acid cleaning 500x


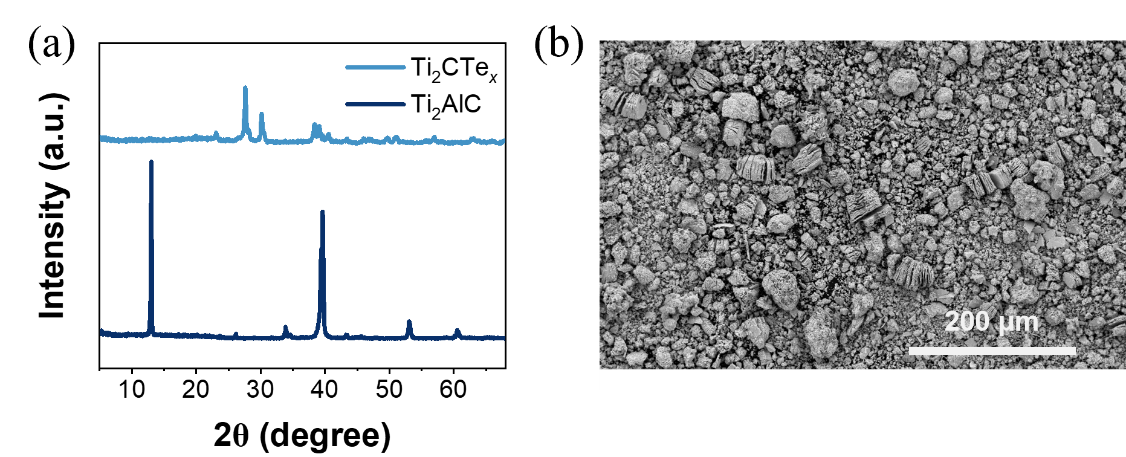


**Fig. S20** Ti_2_CTe*_x_*MXene. **a** XRD before and after etching. **b** SEM macrostructure after acid cleaning 200x


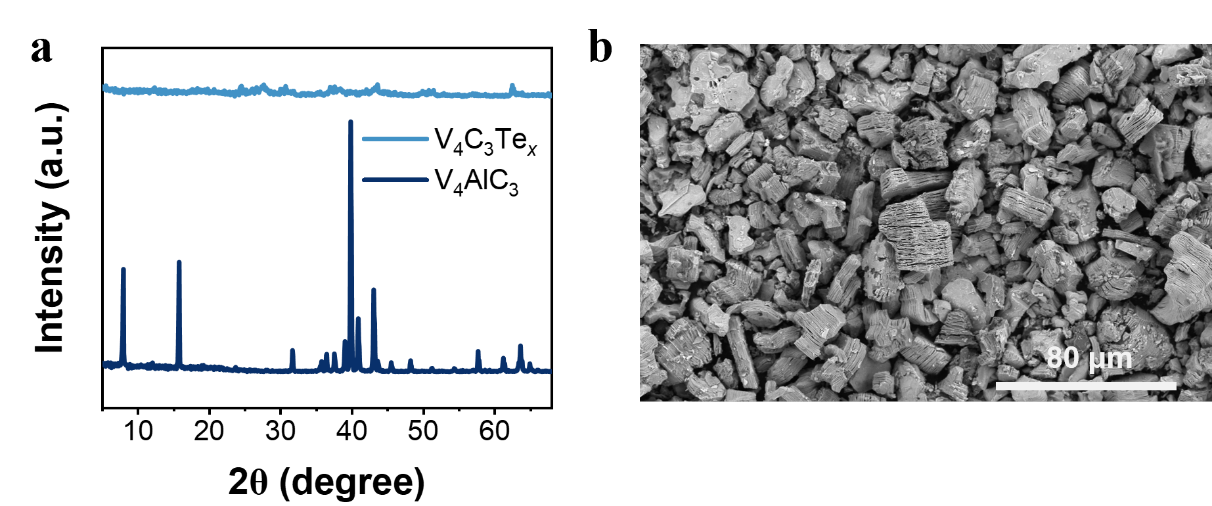


**Fig. S21** V_4_C_3_Te*_x_* MXene. **a** XRD before and after etching. **b** SEM macrostructure after acid cleaning 500x


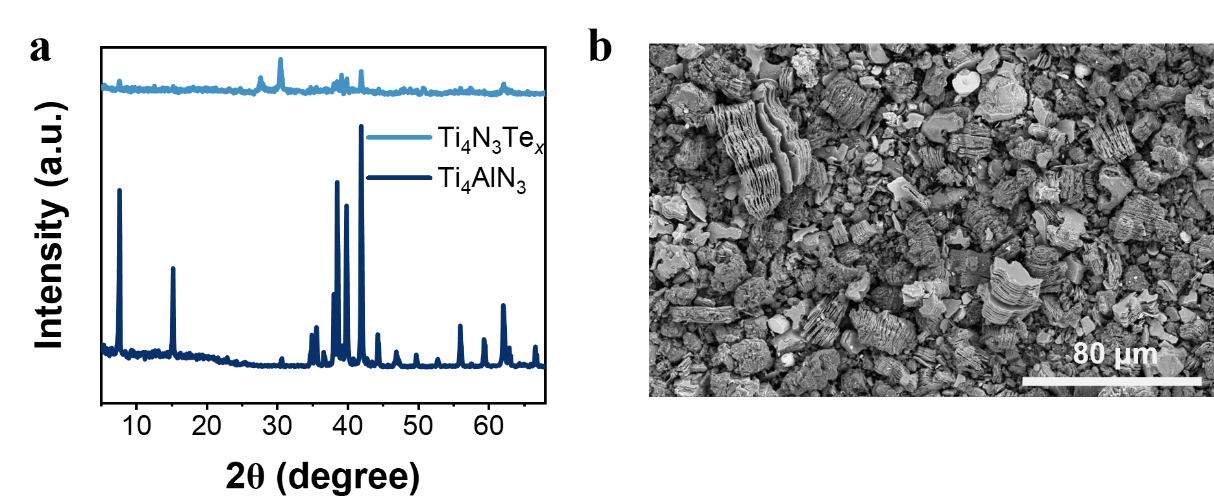


**Fig. S22** Ti_4_N_3_Te*_x_* MXene. **a** XRD before and after etching. **b** SEM macrostructure after acid cleaning 500x


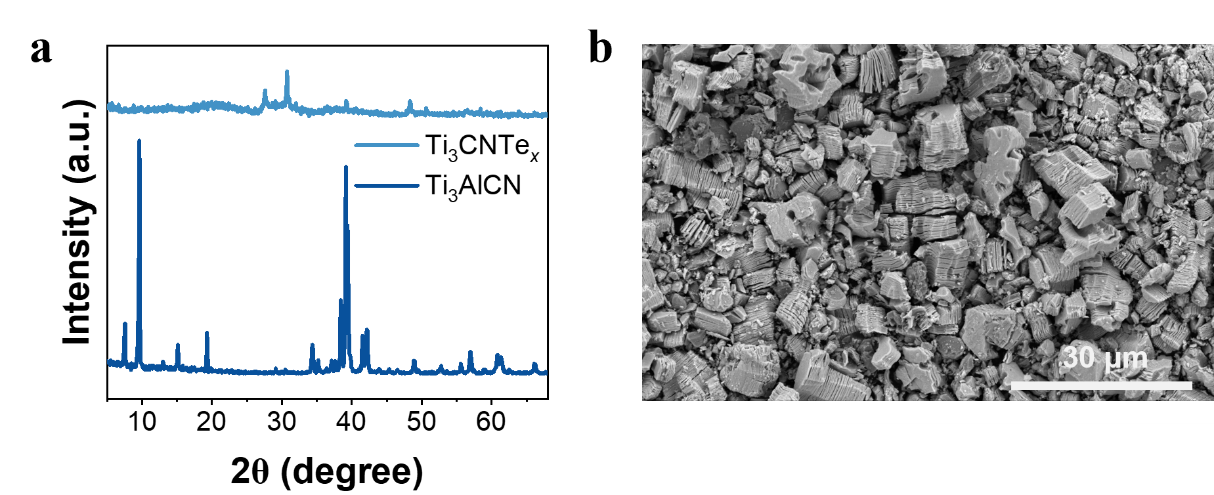


**Fig. S23** Ti_3_CNTe*_x_* MXene. **a** XRD before and after etching. **b** SEM macrostructure after acid cleaning 1000x


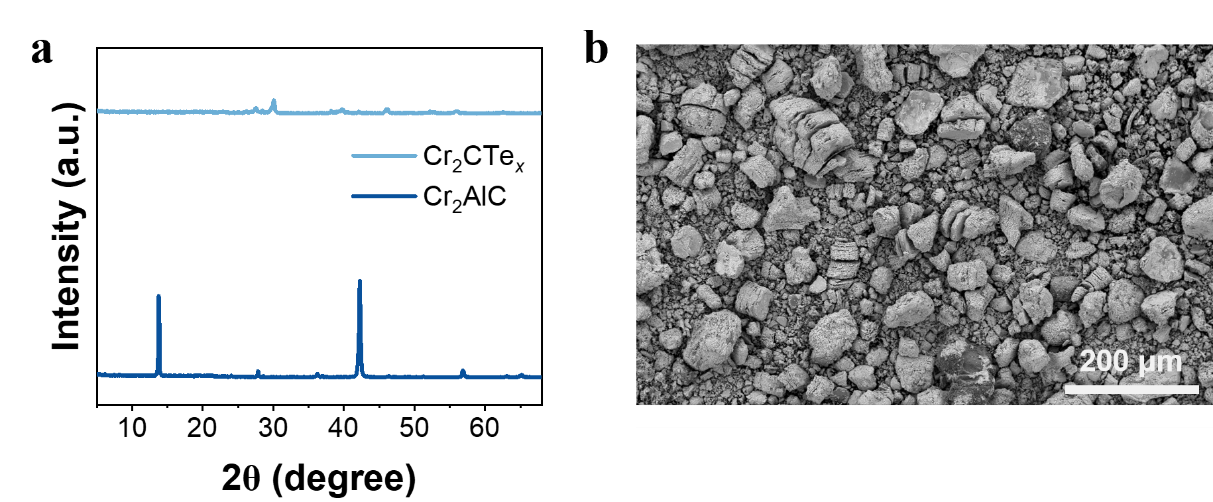


**Fig. S24 C**r_2_CTe*_x_* MXene. **a** XRD before and after etching. **b** SEM macrostructure after acid cleaning 150x


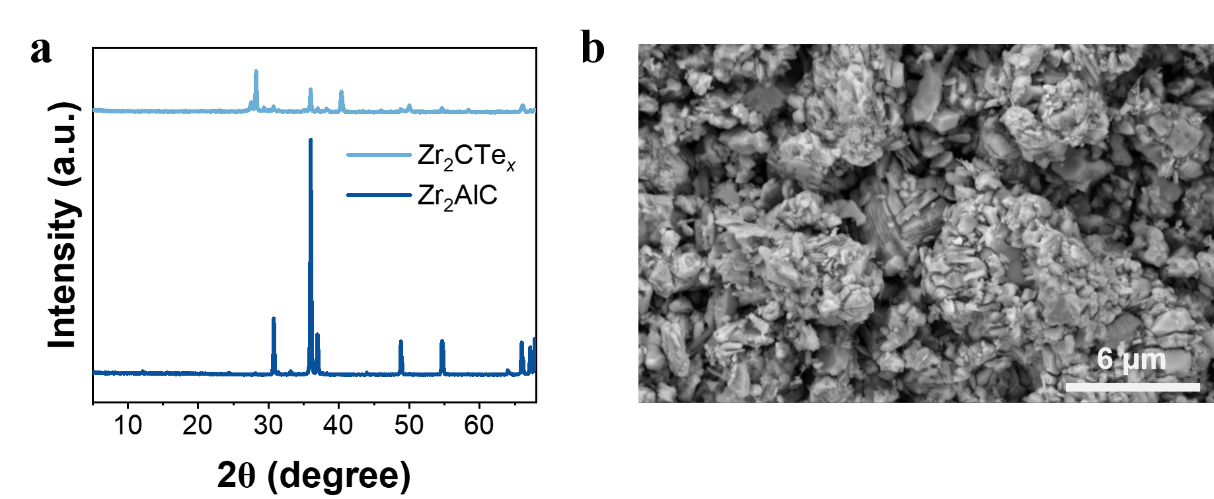


**Fig. S25** Zr_2_CTe*_x_* MXene. **a** XRD before and after etching. **b** SEM macrostructure after acid cleaning 5000x

**Table S7** Types of etching MAX and process parameters

| Raw materials | proportion | Temperature-Time |
| --- | --- | --- |
| Ti_3_AlC_2_-Te | 1:3 | 700- 1h |
| V­_2_AlC-Te | 1:2 | 700-1h |
| Nb_2_AlC-Te | 1:2 | 700-1h |
| Ti_2_AlC-Te | 1:2 | 700-1h |
| Ti_2_AlN-Te | 1:2 | 700-1h |
| Ti_3_AlCN-Te | 1:3 | 700-1h |
| Ta_2_AlC-Te | 1:2 | 700-1h |
| Ti_4_AlN_3_-Te | 1:3 | 700-1h |
| V_4_AlC_3_-Te | 1:3 | 700-1h |
| Cr_2_AlC-Te | 1:2 | 700-1h |
| Zr_2_AlC-Te | 1:2 | 700-1h |


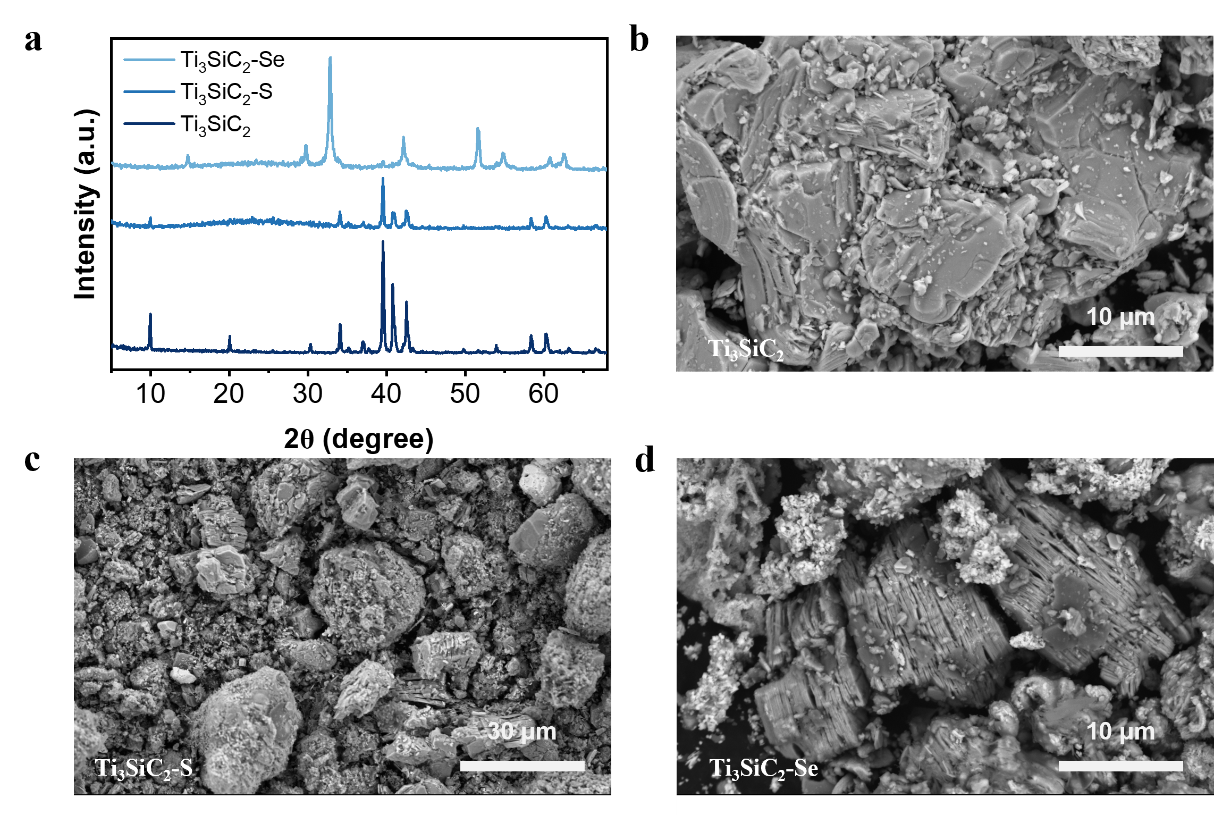


**Fig. S26** Elemental etch extension, **a** Se, S etched Ti_3_SiC_2_ XRD pattern, **b** SEM macrostructure of Ti_3_SiC_2_, **c** SEM macrostructure of S etched Ti_3_SiC_2_, **d** SEM macrostructure of Se etched Ti_3_SiC_2_


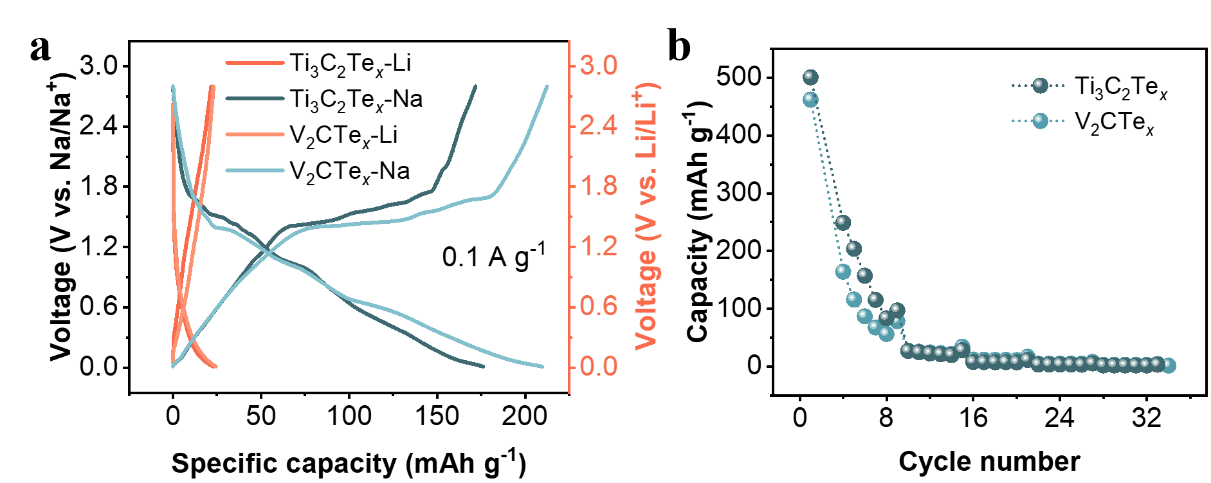


**Fig. S27** **a** Lithium storage and sodium storage charge-discharge curves of Ti_3_C_2_Te*_x_* and V_2_CTe*_x_*, **b** Lithium storage rate performance of Ti_3_C_2_Te*_x_* and V_2_CTe*_x_*


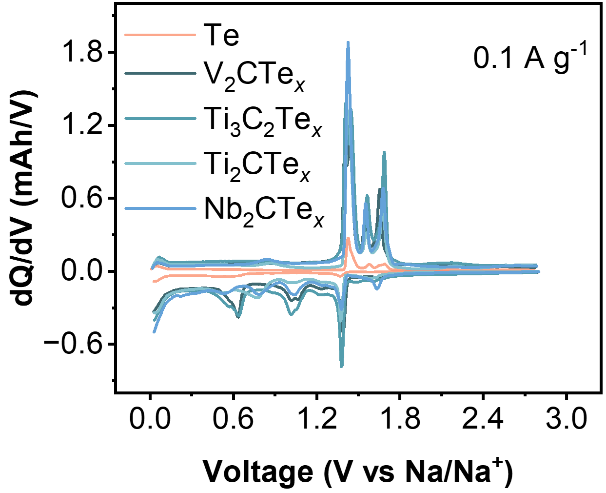


**Fig. S28** Sodium storage dQ/dV curves of different Te-based MXene materials


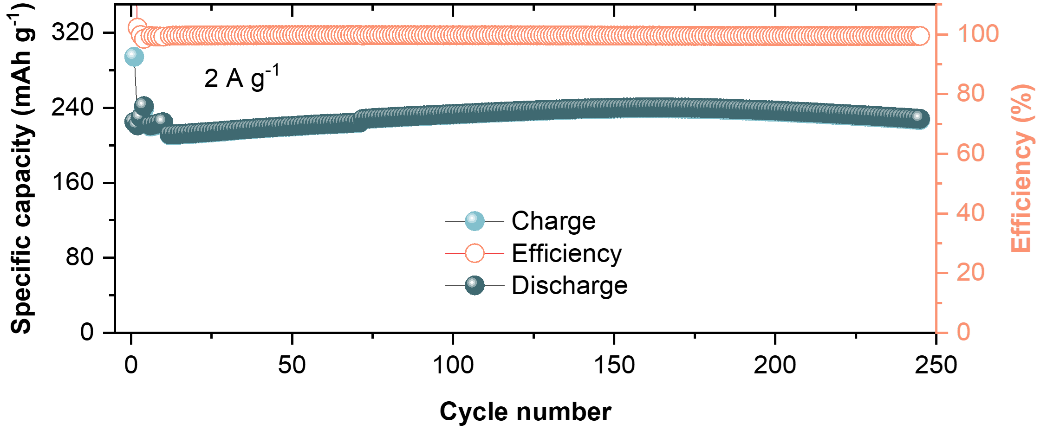


**Fig. S29** V_2_CTe*_x_* MXene Long-cycle performance at 2 A g^-1^


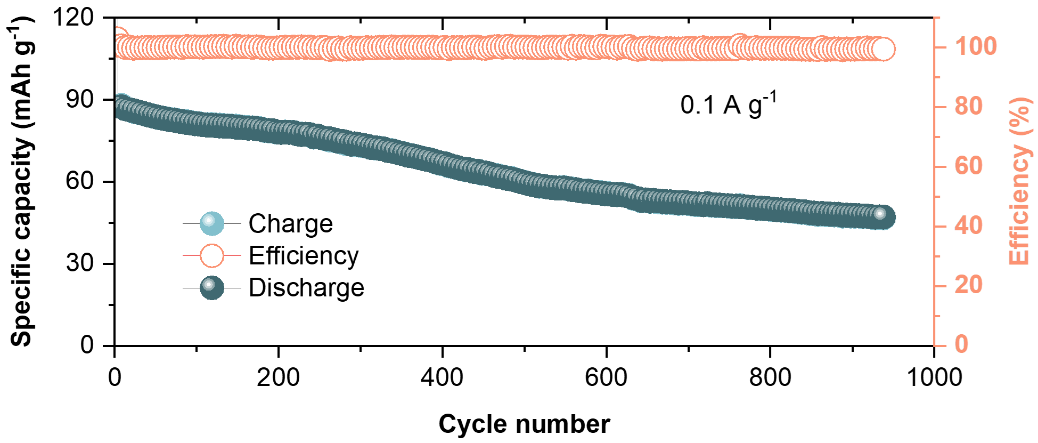


**Fig. S30** Long cycle performance of Te elemental sodium storage


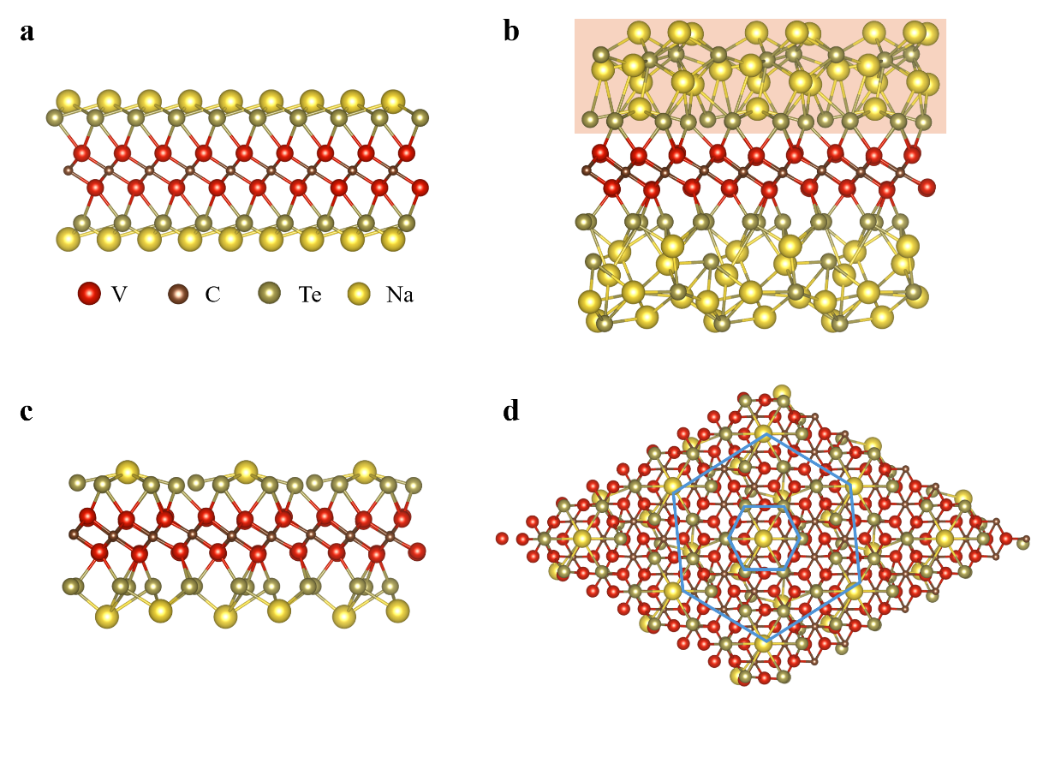


**Fig. S31** DFT theory calculation of V_2_CTe*_x_* sodium storage. **a** Structure before optimization. **b** Structure after optimization. **c** Side view of optimized MXene material surface. **d** Top view of optimized MXene surface


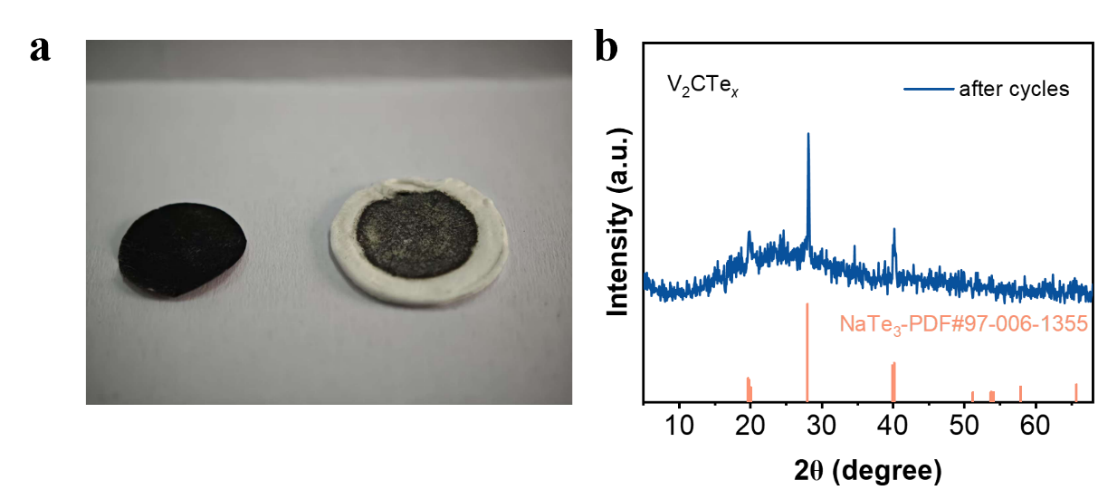


**Fig. S32** V_2_CTe*_x_* electrode after cycling, **a** Disassembled electrode plate and separator, **b** XRD of separator
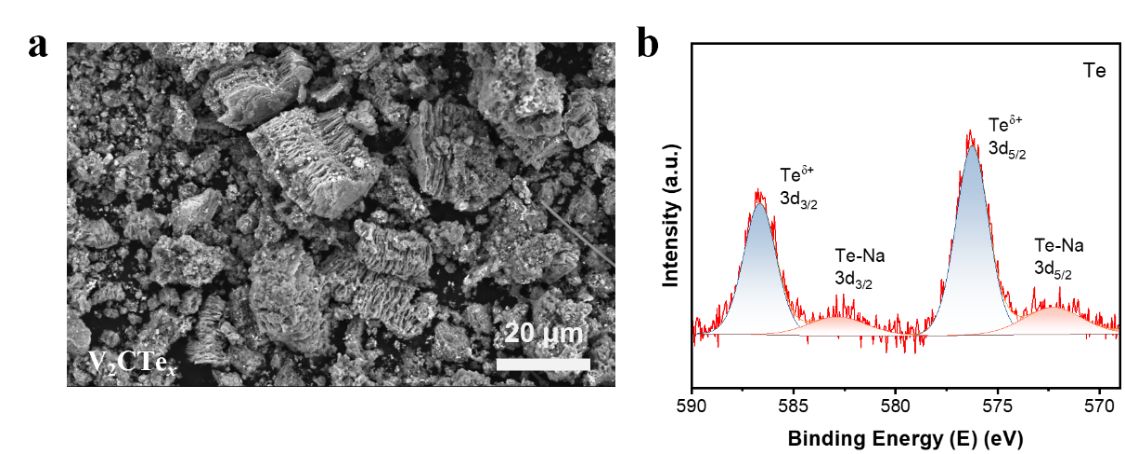


**Fig. S33** V_2_CTe*_x_* electrode after cycling, **a** Microstructure of electrode powder, **b** Te spectrum in V_2_CTe*_x_* electrode XPS


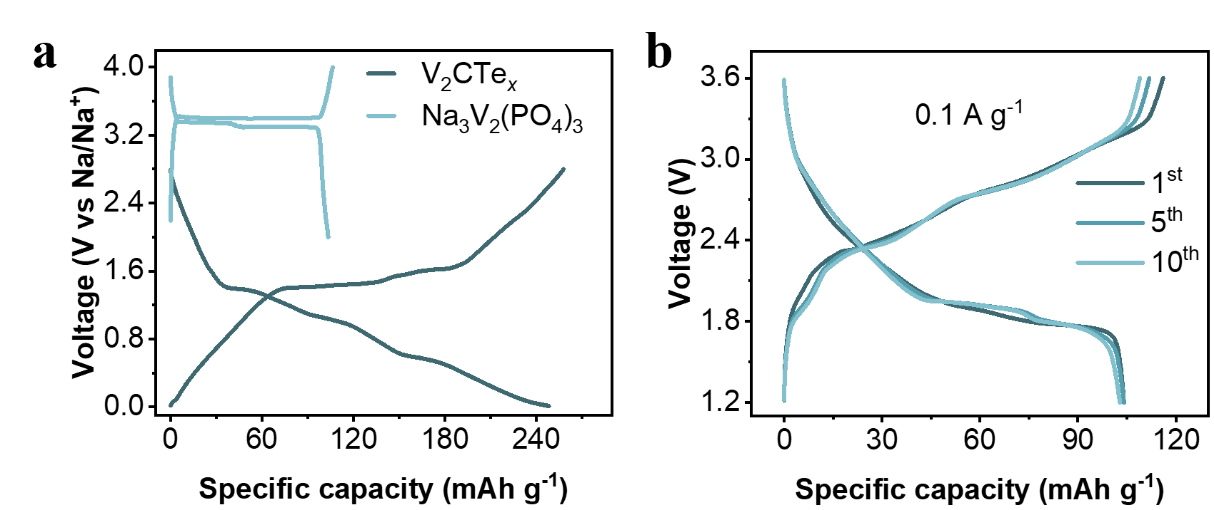


**Fig. S34** Full cell data, **a** Charge and discharge curves of positive and negative materials for half cells, **b** Full cell cycle Data

**Supplementary References**

1. P. Blöchl, Projector augmented-wave method. Phys. Rev. B **50**(24), 17953–17979 (1994). <https://doi.org/10.1103/physrevb.50.17953>
2. Y. Li, H. Shao, Z. Lin, J. Lu, L. Liu et al., A general Lewis acidic etching route for preparing MXenes with enhanced electrochemical performance in non-aqueous electrolyte. Nat. Mater. **19**(8), 894–899 (2020). <https://doi.org/10.1038/s41563-020-0657-0>
3. J. Zhu, S. Zhu, Z. Cui, Z. Li, S. Wu et al., Solvent-free one-step green synthesis of MXenes by “gas-phase selective etching”. Energy Storage Mater. **70**, 103503 (2024). <https://doi.org/10.1016/j.ensm.2024.103503>
4. H. Ding, Y. Li, M. Li, K. Chen, K. Liang et al., Chemical *Scissor*-mediated structural editing of layered transition metal carbides. Science **379**(6637), 1130–1135 (2023). <https://doi.org/10.1126/science.add5901>
5. V. Kamysbayev, A. S. Filatov, H. Hu, X. Rui, F. Lagunas, et al.Covalent surface modifications and superconductivity of two-dimensional metal carbide MXenes. Science, **369**(6506)**:** 979–983 (2020**).** <https://www.science.org/doi/10.1126/science.aba8311>
